# Diversity of the cell-wall associated genomic island of the archaeon *Haloquadratum walsbyi*

**DOI:** 10.1186/s12864-015-1794-8

**Published:** 2015-08-13

**Authors:** Ana-Belen Martin-Cuadrado, Lejla Pašić, Francisco Rodriguez-Valera

**Affiliations:** Evolutionary Genomics Group, Departamento de Producción Vegetal y Microbiología, Universidad Miguel Hernández, Apartado 18, San Juan de Alicante, Alicante, Spain; Department of Biology, Biotechnical Faculty, University of Ljubljana, Večna pot 111, 1000 Ljubljana, Slovenia

**Keywords:** Cell surface glycoproteins, S-layer, Cell-wall, Environmental fosmid library, *Haloquadratum walsbyi*

## Abstract

**Background:**

*Haloquadratum walsbyi* represents up to 80 % of cells in NaCl-saturated brines worldwide, but is notoriously difficult to maintain under laboratory conditions. In order to establish the extent of genetic diversity in a natural population of this microbe, we screened a *H. walsbyi* enriched metagenomic fosmid library and recovered seven novel version of its cell-wall associated genomic island. The fosmid inserts were sequenced and analysed.

**Results:**

The novel cell-wall associated islands delineated two major clades within *H. walsbyi*. The islands predominantly contained genes putatively involved in biosynthesis of surface layer, genes encoding cell surface glycoproteins and genes involved in envelope formation. We further found that these genes are maintained in the population and that the diversity of this region arises through homologous recombination but also through the action of mobile genetic elements, including viruses.

**Conclusions:**

The population of *H. walsbyi* in the studied saltern brine is composed of numerous clonal lineages that differ in surface structures including the cell wall. This type of variation probably reflects a number of mechanisms that minimize the infection rate of predating viruses.

**Electronic supplementary material:**

The online version of this article (doi:10.1186/s12864-015-1794-8) contains supplementary material, which is available to authorized users.

## Background

The large diversity of genes present in the pangenome of prokaryotic species (both bacteria and archaea) has been one of the most remarkable revelations of genomics. In addition to a ‘core genome’ that contains genes present in all strains, genomes can harbour a number of ‘flexible’ genes that are absent from one or more strains or that are unique to each strain [1]. It is now well established that the flexible genome is a major contributor to the genetic diversity of prokaryotic communities. It is often organized into large clusters of genes known as flexible genomic islands (fGI) [2]. One way to explore the species pangenome is to conduct a comparative genomic study of multiple isolates. However, this type of study is very difficult to achieve with microbes that are not readily cultivated, such as *Haloquadratum walsbyi*. This halophilic archaeon represents up to 80 % of cells in NaCl-saturated brine of most saltern crystallizers [3, 4] and saturated brines worldwide (e.g., [5–7]) but is notoriously difficult to maintain under laboratory conditions. Consequently, only two strains are available in pure culture strain HBSQ001, isolated from crystallizer CR30 in a Santa Pola saltern in Spain [8] and strain C23, from Geelong saltern in Victoria, Australia [9].

The issues raised by cultivation can be circumvented through metagenomics. For example, fGI can be delineated through tiling of metagenomic reads from the microbe’s habitat to its reference genome. In such an experiment, known as genomic recruitment, the well-represented “core” genes will recruit a large number of metagenomic fragments at high sequence similarity while, at the same time revealing areas of underrepresented flexible genes. Previously, some of us applied this approach to describe variability in natural population of *H. walsbyi* in the CR30 metagenome [10]. Recruitment of *H. walsbyi* HBSQ001 genome in this metagenome showed that the *H. walsbyi* population was composed of multiple clonal lineages that differ in four genomic regions. This variability was attributed to i) genes involved in formation of cellular wall including surface layer (S-layer; genomic island 1, GI1), ii) genes involved in uptake of nutrients (genomic islands 2 and 4, GI2 and GI4) and iii) provirus integration (genomic island 3, GI3) [10]. Variable islands were later revealed by comparison with the genome of the Australian isolate C23 [11] and similar situations were found for many bacteria (for a review see [12]), what makes this scenario scalable to prokaryotes at large. In free-living prokaryotes, the flexible genes are considered to contribute to organismal fitness. For example, the variability at the level of S-layer is considered a strategy to dilute predating pressure [13, 14].

Alternative views were reported, most recently in a recruitment study on environmental genomes of *H. walsbyi* assembled from metagenomic DNA of Lake Tyrell. This study reported GI2 but not the other genomic islands and observed large genomic rearrangements, insertions and deletions [15].

Given the current state-of-the art, it is difficult to attribute the majority of bacterial and archaeal losses to predating protist grazers. In crystallizers of studied Spanish salterns the grazers appeared only occasionally and were not actively feeding on bacteria and archaea [16, 17]. However, they have been reported from other hypersaline systems (e.g., [18]) and were found to actively feed on bacteria and archaea in the crystallizers of a Korean saltern [19]. On the other hand, viral communities reach unusually high numbers (up to 10^9^ per ml) in crystallizers [17, 20, 21]. Intriguingly, in the system studied here, viruses do not exert strong control over growth and abundance of bacteria and archaea and cause less than 5 % of cell lyses per day [17] and in 40 years of studies on crystallizer CR30, a massive lysis event collapsing the *H. walsbyi* population has never been observed. In crystallizers of salterns located in some parts of the world where the variation in environmental factors through time is negligible and *H. walsbyi* is the dominant species (e.g., CR30 of Santa Pola) this seems to be the pattern. But it is possible that hypersaline systems that are subjected to variation of environmental factors may behave differently. For example, Australian Lake Tyrell experiences summer desiccation, accompanied by a change in solution chemistry and significant variation in temperature. Not surprisingly, the structures of the archaeal, bacterial and viral populations were found to change on timescales of months to years [22, 23] and these changes correlated with changes in environmental factors. However, even in such dynamic systems the viral diversity remained constant [24].

To extend our knowledge on population genomics in *H. walsbyi*, we decided to use metagenomics to recover flexible genomic islands directly from environmental DNA by fosmid cloning. We have focused our study to the area of GI1, which is enriched in cell-wall related genes that include the S-layer. This island stretches over 45.4 kb in strain HBSQ001 and 8.2 kb in C23 [10, 11]. Burns et al., [25] reported that the cell wall was three-layered in strain HBSQ001 and two-layered in strain C23. This difference was hypothesized to be a consequence of the different GI1 content and remains to be established experimentally [11]. We retrieved and studied GI1 of seven previously unknown *H. walsbyi* lineages that were present in the CR30 crystallizer at a single sampling time. In addition to provide a detailed description of these novel regions, we discuss their contribution to our current understanding of population genomics of this archaeal species.

## Results

### General features of the metagenomic fosmid inserts

Three versions of *H. walsbyi* GI1 are presently available, two in the genomes of *H. walsbyi* C23 and HBSQ001 and one present in fosmid clone eHwalsbyi559 [10]. The last two came from the same pond, crystallizer CR30, which has been extensively studied for many years [26]. In order to recover other environmental versions of GI1, we made use of the fosmid library FLAS CR30-2002 that was constructed in 2002 (just two years after the sample used for isolating the strain HBSQ001) using CR30 environmental DNA [27]. Within the fosmid-end sequences of this library, we found seven clones whose inserts overlapped GI1 in the genomes of HBSQ001 and C23 (Additional file 1). The clones were recovered and their inserts completely sequenced and assembled. In accordance with existing nomenclature [10], the inserts were denominated eHwalsbyiGI1_1 to eHwalsbyiGI1_12.

The fosmid inserts ranged from 35.9 to 41.9 kb in length and from 47 to 53 % in GC content and encoded a total of 185 putative ORFs (Table 1). Most of the obtained sequences had no identical counterparts in databases and despite intensive annotation efforts (see Methods), 40 % of the ORFs were hypothetical proteins. In a general classification in clusters of orthologous groups (COGS) (Additional files 2 and 3), only 37 % of the ORFs could be assigned and a significant portion (12.9 %) belonged to the class transposase and inactivated derivatives, highlighting the dynamic nature of eHwalsbyiGI1. Also abundant were categories (P)-inorganic ion transport and metabolism (7.1 %) and (M) cell wall/membrane biogenesis (7.1 %). ORFs affiliated with these two groups corresponded exclusively to ABC-type cobalt/Fe^3+^ transport system (periplasmic component) and S-layer domain proteins, both of which are typically found in GI1 of *H. walsbyi* and similar genomic islands of other species [28]. Detailed information on the putative functions of the 185 ORFs, their putative post-translational modifications and cellular localisation are shown in Additional file 4.

**Table 1:** General features of fosmid inserts presented in this study

Fosmid characteristic	eHwalsbyiGI1 number						
	1	4	5	6	7	9	12
Insert size (kb)	37.0	41.9	37.5	36.9	35.9	35.9	33.8
GC-content (%)	48.51	50.04	47.06	47.41	52.64	48.31	49.71
Number of ORFs	31	25	16	31	22	32	28
Number of hypothetical proteins	16	6	4	14	9	20	5
Number of COG-assigned ORFs	15	11	10	12	6	5	9
Classification	*H. walsbyi*	*H. walsbyi*	*H. walsbyi*	*H. walsbyi*	*H. walsbyi/ halovirus*	*H. walsbyi*	*H. walsbyi*

**Fig. 1:**
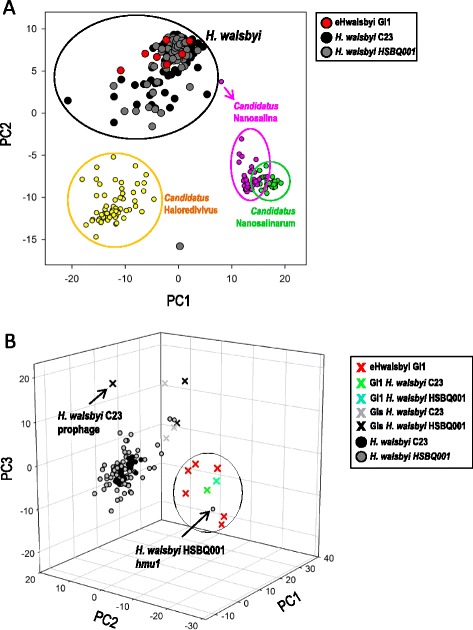
Principle component analysis of tetranucleotide frequencies usage in **a**) eHwalsbyiGI1s (red), *H. walsbyi* HBSQ001 (grey), C23 (black), *Candidatus* Haloredivivus sp. G17 (yellow), *Candidatus* Nanosalina sp. J07AB43 (purple) and *Candidatus* Nanosalinarum sp. J07AB56 (green) and **b**) eHwalsbyiGI1s, and *H.walsbyi* HSBQ001 genomic segments of 35 kb in size

To confirm that these DNA fragments belonged to uncultured *H. walsbyi* and not to other Archaea also present in the brines, we calculated their tetranucleotide frequencies and performed a principle component analysis [29]. When DNA fragment size is appropriate, the oligonucleotide frequencies carry a good phylogenetic signal useful to identify species or genera of environmental DNA fragments [30]. Therefore, we first compared tetranucleotide usage in eHwalsbyiGI1s, genomes of *H. walsbyi* and genomes of other CR30 microbes with low GC-content (Fig. 1a). This analysis showed that all the eHwalsbyiGI1s sequenced cluster together with the genomes of *H. walsbyi*. In a second analysis, we compared the eHwalsbyiGI1s and *H. walsbyi* genomes split into non-overlapping fragments of an approximate size of GI1 (35 Kb). This analysis clustered eHwalsbyiGI1s, isolate HBSQ001 and C23 GI1s and the giant protein halomucin separately from the ‘core’ genomic regions of *H. walsbyi* genomes (Fig. 1b). This reflects the particular composition of these proteins which are often enriched in acidic aminoacid residues and contain few cysteine residues [31], as observed in eHwalsbyiGI1s (Additional file 5). Another hallmark of surface-expressed proteins is the abundance of amino acid repeats. The repeats are presumed to allow surface proteins flexibility in structure and to bind water, ions and other substrates [30]. In eHwalsbyiGI1s, repeats (2-62 amino acids in length) were found in 78 out of 185 ORFs studied (Additional file 6). Among 46 unique repeats detected, 26 were enriched in proline and threonine (PT-repeat). This type of repetition was found in genes encoding cell surface glycoproteins, S-layer proteins, and subtilisin-like serine proteases. In eleven proteins, this repeat was a simple direct PT-repeat (5 to 29 times), while more complex PT-repeats were most often unique to individual genes (the most complex being TS[TP,5]SPTPSPTATPTQ[TP,3]LPTPAPTNT[P,3]TSA[PT,2]A[TP,2]TL[TP,3] found in eHwalsbyiGI1_1_30 cell surface glycoprotein) (Additional file 6). Repeat T[E,2]TATPEPTAT[E,2]P was common to S-layer protein genes of eHwalsbyiGI1_5, eHwalsbyiGI1_6 and eHwalsbyiGI1_12. The S-layer protein genes had another common repeat, the pentapeptide AVGDL. The exact localization of the repeats in the eHwalsbyiGI1 and eHwalsbyi559 [10] genes is shown in Additional file 6.

### Gene order and regions of synteny between fosmid clones

**Fig. 2:**
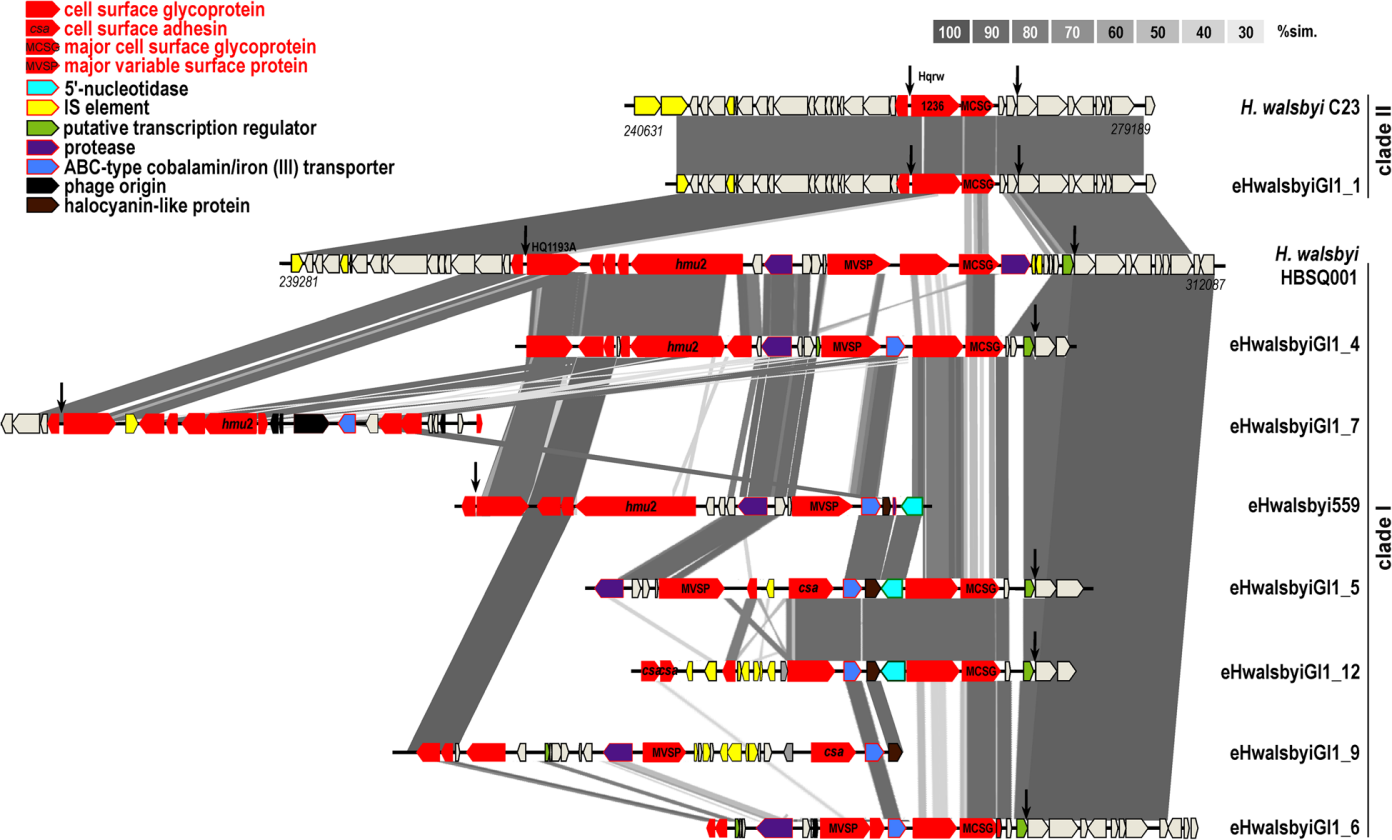
GI1s of *H. walsbyi* isolates HBSQ001 and C23, eHwalsbyi559 and eHwalsbyiGI1s obtained in this study. The shadings link homologous regions between the GI1s and eHwalsbyiGI1s and percent identity is shown in the scale located in the upper right corner. Locations of G1s on *H. walsbyi* genomes are indicated by nucleotide position numbers at the beginning and at the end. Arrows indicate the origin and end of the GI1. ORF names are as annotated in GenBank, are colour coded and designated on the left

The TBLASTX comparisons of the novel eHwalsbyiGI1s, eHwalsbyi559 and their equivalent regions in *H.walsbyi* HBSQ001 and C23 delineated two major clades (Fig. 2). Clade I was formed by eight sequences. These were all significantly different (≤85 % identity) and presented mosaic structure in which clusters of conserved genes alternated with highly variable ones. No gene duplications or inversions could be observed. The largest regions of synteny were observed between eHwalsbyiGI1_5 and 12 and genomic sequences of *H. walsbyi* HBSQ001, eHwalsbyi559 and eHwalsbyiGI1_4. The most different fosmid insert analysed was eHwalsbyiGI1_7. The lack of synteny was consistent with a 16 kb region within the island deriving from an inserted provirus (see below). In contrast, Clade II was composed of completely syntenic and almost identical (≥97 % identity) sequences (eHwalsbyiGI1_1 and the equivalent region in *H. walsbyi* C23). It is remarkable that, in spite of their high similarity, these sequences originate from geographically distant salterns (Spain and Australia) that were sampled at different time-points (2002 and 2004) [9, 27].

### Overview of genes encoding cell surface glycoproteins

The most remarkable feature of eHwalsbyiGI1s was the presence of unique clusters of genes encoding cell surface proteins. Only two of these genes were common to all eHwalsbyiGI1s, likely because most fosmid sequences did not contain full-length versions of GI1. The first is located to the 5' end of GI1 (HQ1193A in HBSQ001, Hqrw_1236 in C23 and its homologs in eHwalsbyiGI1s). It encodes a putative cell surface protein with an N-terminal signal peptide and presumed to be anchored in the cell membrane through a C-terminal transmembrane helix. Its amino acid sequence is well conserved at both terminuses (N-terminal, 65–100 % similarity (~220 amino acids); C–terminal, 75–100 % similarity (~900 amino acids)). The two conserved regions are separated by a proline-threonine repeat-motif that among the different homologs ranges from 110 to 250 amino acids and contributes significantly to their variability. A similar phenomenon has been observed previously in cell surface proteins of certain pathogenic bacteria [32].

**Fig. 3:**
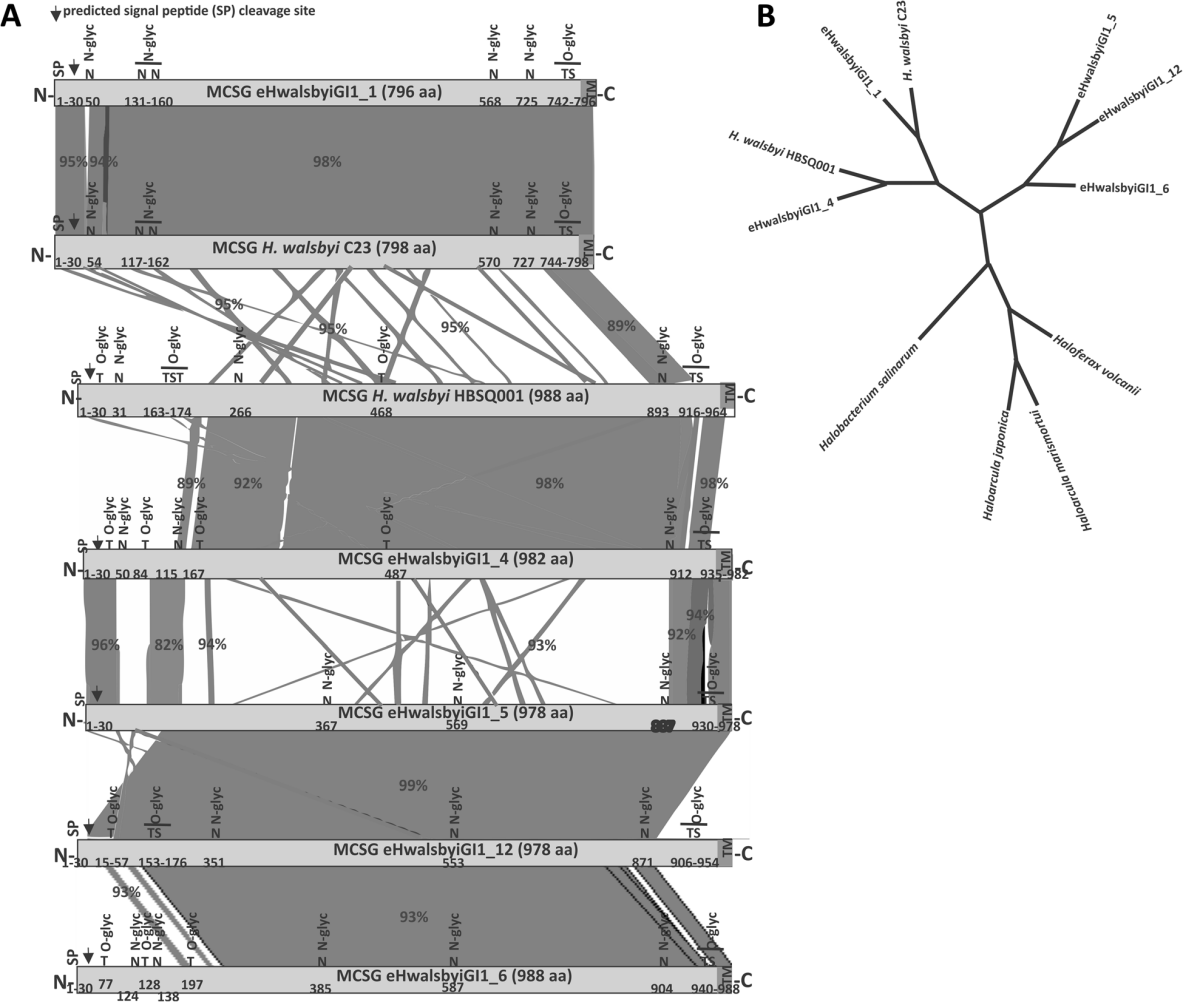
**a** Schematic alignment of genes annotated as major cell surface glycoprotein (S-layer). The shadings link homologous regions between the S-layer genes and percent identity is showed in numbers. TM- transmembrane helix, N-glyc and O-glyc indicate putative glycosylation sites. Numbers indicate positions of N-glycosylation. Arrows indicate predicted site of lipid modification. **b** Maximum likelihood tree showing the phylogenetic relationship between *H. walsbyi* and eHwalsbyiGI1 genes annotated as major cell surface glycoprotein and related genes in other halophilic archaea

The second common gene is less conserved in terms of amino acid sequence (45 to 98 % similarity) and corresponds to the major cell surface glycoprotein (MCSG; HQ1207 in HBSQ001; Hqrw_1237 in C23 genome). We know from previous studies that this gene is expressed in *H. walsbyi* HBSQ001 and C23, and that it encodes the S-layer protein that is exposed on the surface of *H. walsbyi* cells as monomolecular crystalline array [11, 33]. All these homologous genes are uniform in size (~900 amino acids) and similar in terms of domain organisation and post-translational modifications. Interpro searches predicted an N-terminal signal peptide, followed by major cell surface protein family signature (IPR026458), and a recognition sequence for an archaeosortase, an enzyme that in related species mediates proteolysis-coupled, covalent cell surface attachment [34]. Besides, they contain regions that are putatively N-glycosylated and a threonine rich O-glycosylated carboxy region, as illustrated in Fig. 3a. The remarkable diversity of these proteins is reflected in their phylogeny as they form three distinct groups (Fig. 3b).

Among the highly variable genes, the most striking are the four homologs of halomucin 2 gene, *hmu*2 (30–75 % similarity). Previous studies suggested that Hmu2 contributes to the formation of a capsule in strain HBSQ001 [35]. Interpro server predicted these homologs to encode large (1350–3072 amino acids) multidomain extracellular proteins with domains involved in recognition/depolymerisation of polysaccharides: N-terminal pectin lyase fold/virulence factor, parallel beta helix repeat domains, and acidic peptide-dependent hydrolase/peptidase domain. Besides, the homologs also contained N-terminal PapD-like domains, which can contribute to assembly of surface organelles involved in cellular attachment [36]. Whether these proteins can contribute to the formation of capsule or aid in aggregation of individual *H. walsbyi* cells into the postage stamp-like sheet, remains to be established experimentally.

A spectrum of carbohydrate-recognizing protein domains is the common attribute of the only group of genes that is conserved in terms of synteny. The domain structure and the amount of variability in these proteins is reminiscent of the one in surface antigens of pathogenic bacteria where it reflects strategies to avoid host immune response (e.g., [37]). These genes, annotated as major variable surface proteins (HQ1205 and its homologs in eHwalsbyi559, eHwalsbyiGI1_4, 5, 6, and 9; 32–52 % similarity) encode putative integral membrane proteins that vary in length (1260–1626 amino acids) and in domain architecture. They present either a pectin lyase fold/carboxypeptidase/parallel beta-helix repeat or an invasin-intimin/cell adhesion domains/bacterial immunoglobulin-like domains. Furthermore, the C-terminal region of HQ1205 and homologs in eHwalsbyi559, eHwalsbyiGI1_5 and 6 has a Ca^2+^ binding domain.

### ORFs involved in metabolic activities

Clade I sequences contain genes predicted to encode extracellular proteases that are anchored in the membrane by their C-terminal region (with the exception of eHwalsbyiGI1_12). The predicted proteins possess an EF hand motif, a helix-loop-helix motif found in a large family of Ca^2+^ binding proteins. Besides, Clade I gene clusters (with the exception of GI1 of *H. walsbyi* HBSQ001) contain an ABC cobalt/iron (III) transporter gene. Meanwhile, eHwalsbyi559, eHwalsbyiGI1_5 and 9 contain a 5' nucleotidase gene. Inserts of eHwalsbyi559, eHwalsbyiGI1_5, 9 and 12 contain homologs (50–99 % similarity) with cupredoxin domains typically found in archaeal type I copper peripheral membrane proteins. Such proteins, termed halocyanins, serve as mobile electron carriers and are commonly found in halophilic archaea; in fact, the core genomes of *H. walsbyi* strains contain three halocyanins. However, the similarity of these core genomic halocyanins to those in eHwalsbyiGI1s is restricted to domain organisation and gene size and their similarity to database sequences is ≤30 %.

### Mobile genetic elements

In contrast to GI1 of *H. walsbyi* HBSQ001 and C23 [10, 11], the eHwalsbyiGI1s contain mobile genetic elements: ISH9-type (eHwalsbyiGI1_5), IS-1341 (eHwalsbyiGI1_7), and IS-1341 and IS605 transposases (eHwalsbyiGI1_ 9 and 12). Their presence is associated with a decrease in synteny, which is most evident in the transposase-rich eHwalsbyiGI1_9 and eHwalsbyiGI1_12. This indicates that mobile genetic elements contribute to the dynamic character of this region.

In eHwalsbyiGI1 there is some evidence that *H. walsbyi* expands its pangenome through provirus integration. The sequence of eHwalsbyiGI1_7 harbours proviral remnants downstream to the gene homolog of HBSQ001 HQ1196A (Fig. 2). It contains two hypothetical proteins that are similar to hypothetical proteins in environmental halophages eHP-12 (37 %) and eHP-1 (96 %). Further downstream, a putative primase is similar to *Halorubrum* virus C6phi46 (31 %). The GC content of the region affiliated with the virus was 54.3 % and similar to that of virus HRPV-1 (54.2 %), a representative of the haloarchaeal pleomorphic viruses [38, 39].

### Recombination events contribute to the diversity of cell-wall associated genomic island 1

**Fig. 4:**
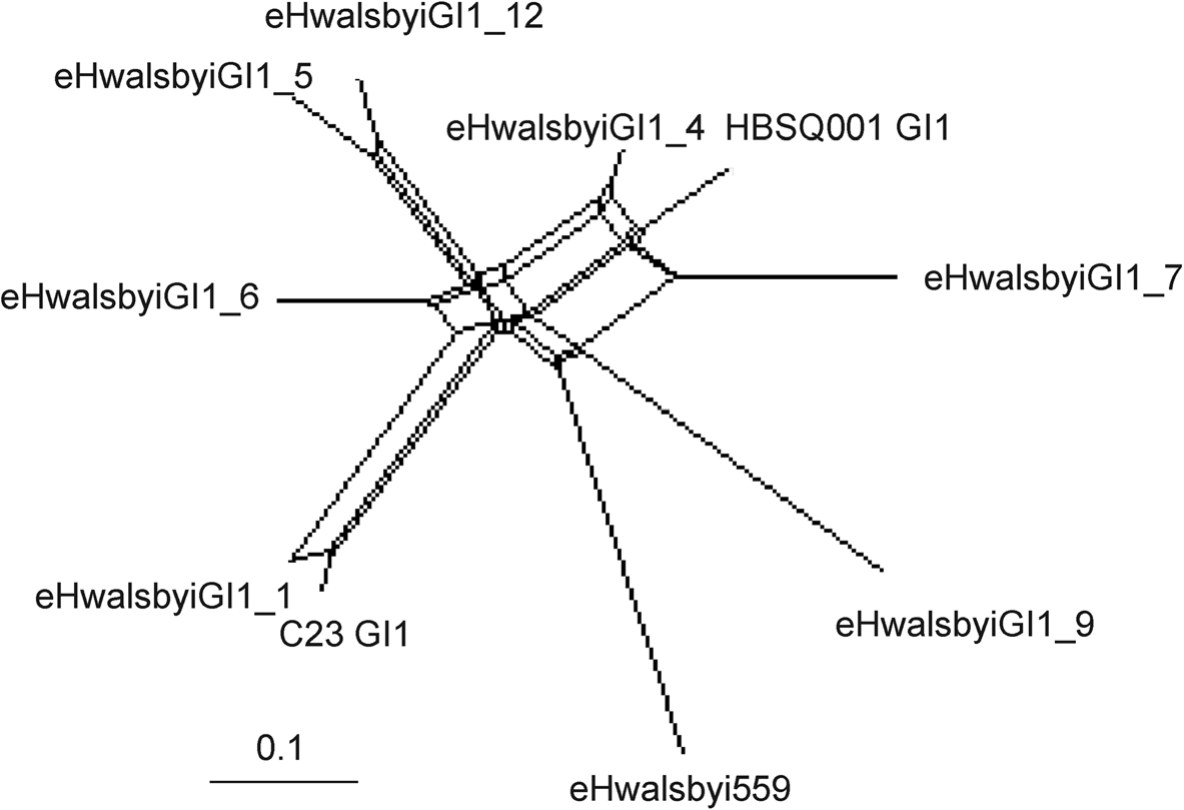
Phylogenetic network of full length GI1 of *H. walsbyi* HBSQ001 and eHwalsbyiGI1s sequences created with Splits Tree using the uncorrected P characters transformation

Previous studies indicated that homologous recombination contributes to diversity of cell-wall associated genomic islands in aquatic prokaryotes, including *H. walsbyi* (e.g., [40, 41]). This prompted us to analyse recombination events among the eHwalsbyiGI1 and equivalent regions in the sequenced *H. walsbyi* genomes using SplitsTree [42]. This tool detected conflicting phylogenetic signals within the sequences which resulted in a reticulate phylogenetic tree (Fig. 4). PHI test for recombination confirmed that the conflicting signals were due to recombination (test statistic *Φ* = 0.000).

The alignments were further analysed using RDP4 to identify the positions of recombination breakpoints. RDP4 detected 13 recombination events that were distributed along eight out of the ten genomic islands studied (Additional file 7). In the case of eHwalsbyiGI1_6 and 9, the recombined segments stretched throughout most of the island length (along 25 kb and 21 kb, respectively) and included the gene encoding the S-layer protein. In a similar fashion, a relatively small (about 250 bp) recombination event affected the S-layer protein in fosmid eHwalsbyiGI1_1. In fosmid eHwalsbyiGI1_7, a recombinational event affected the homologue of HQ1193. All these recombinations were identified by more than two methods, were highly supported (P-values ≤10^-70^, Additional file 7) and indicate that genetic exchange of cell-wall associated gene clusters in *H. walsbyi* could have an effect similar to that of capsular switching in pathogens. As one would expect, the recombination events were not limited to the region affiliated with GI1, but extended upstream or downstream of this region as evident in eHwalsbyiGI1_1.

### The genes in the cell-wall associated genomic islands are maintained in the population and are under purifying selection

Recently transferred genes are known to be under faster and relaxed evolution and are expected to have high rates of nonsynonymous substitutions to nonsynonymous site (dN) compared to synonymous substitutions to synonymous site (dS) [43]. Over time, the genes that contribute to organismal fitness will accumulate less amino acid changing mutations and this will be reflected in dN/dS ratio that is significantly lower than one. Accordingly, the non-beneficial genes will display a dN/dS ratio near one [44]. However, such values need to be taken cautiously when analyzing closely related genomes (e.g., [45]) as they can arise because the time has been too short for selection to act [46] or because nucleotide substitutions within a species may represent segregating polymorphisms and not fixed differences [47].

The selection analysis was performed using JCoDa [48] and KaKs calculation tool (http://services.cbu.uib.no/tools/kaks) (see Methods). The results were comparable and showed that most homologous genes present in the cell-wall associated genomic islands have dN/dS ≤1 (Additional file 8). This indicates that the genes were not recently acquired, and that they are functional and maintained in the population by selection. In fact, only a few genes, such as HQ1194 and HQ1195, displayed dN/dS ≥1 and could be considered under positive selection. These results are in agreement with values obtained when selection analysis was applied to similar regions in marine and pathogenic microbes [49, 50].

## Discussion

The metagenomic approach used in this study allowed us to study unique genome stretches belonging to seven previously unknown lineages of *H. walsbyi* that simultaneously inhabit crystallizer CR30. The analysis of their gene content confirmed the contribution of this region to the formation of a functional cell wall. However, the different versions of eHwalsbyiGI1 recovered presented some additional interesting features. Particularly notable was the abundance of proteins predicted to have carbohydrate-binding domains. Further analyses of individual eHwalsbyiGI1 genes identified putative proteins that could contribute to the protection of cell from desiccation (e.g., homologs of *hmu*2).

Individual eHwalsbyiGI1 genes were under purifying selection, indicating that they are beneficial to the *H. walsbyi* population. We attributed their high level of variability (average amino acid sequence similarity of 85 %) to multiple processes; such as frequent homologous recombination and the presence of transponible elements. Furthermore, the abundance of repetitive gene regions indicated that *H. walsbyi* achieves additional variability of the cell surface proteins through modification of existing genes. For example, homologs of HQ1193A differ mostly in the length of proline-threonine repeat motif. It is known that repetitive gene regions are prone to errors during replication which generate changes in the number of repeat units that are up to 10000 times more frequent than point mutations [51].

The variability of clonal lineages at the level of cellular envelope is a well-documented phenomenon and is considered to reflect the interplay between archaeal and bacterial cells and their viral predators [10, 14, 52–54]. Hypersaline waters (over 30 % w/v) typically have high concentrations of bacterial and archaeal cells (ca. 10^7^ per ml) and even higher levels of virus particles (ca. 10^9^ particles per ml), while supporting few grazing ciliates and flagellates [17, 18, 55] . We know from previous studies [39], that the *H. walsbyi* population is infected by viruses of the order Caudovirales, which use surface proteins, polysaccharides or lipopolysaccharides as receptors in order to initiate the infection [56], and it is not uncommon for bacterial populations to respond to phage predation by varying their cell surface proteins [57]. Actually, the analysis of eHwalsbyiGI1 genes yielded an impressive arsenal of putative virus defence mechanisms. For example, the presence of capsule-related *hmu*2 gene in several eHwalsbyiGI1s indicates that the corresponding lineages could produce an additional envelope layer. This layer would mask the viral receptor forcing viruses to cleave the capsular polysaccharide in order to access the receptor. In line with these observations, the presence of putative glucanase genes in environmental genomes of halophilic viruses has been reported from this and other environments [24, 39]. Prokaryotic cells can also avoid viral adsorption by modifying the receptor proteins. A well-known example comes from *Prochlorococcus*, in which point mutations on genes involved in the synthesis of the phage receptor (the O-chain polysaccharide encoded in a hypervariable genomic island) originated resistant strains [52]. A similar variation (there are point mutations scattered throughout the gene) can be seen in the GI1 gene that encodes the pseudoperiplasmic component of ABC cobalamin/iron (III) transporter, which displays high level of sequence conservation (≥90 % amino acid similarity). As membrane-bound lipoproteins, these archaeal ABC transporter proteins are possibly targets of phage attachment.

Another edge in viral avoidance can be gained by the presence of multiple proteins on the cellular surface [57]. We know from previous studies that *H. walsbyi* C23 has 12 genes annotated as cell surface (glyco)proteins. However, only four gene products were detected on the cell surface. These correspond to the S-layer protein, a product of GI1’s Hqrw_1237 and products of Hqrw_1240, Hqrw_1641 and Hqrw_2184 that are located elsewhere in the genome and are common to both isolates [11]. In contrast, the cells of *H. walsbyi* HBSQ001 lack CRISPR but have the potential to build a more complex cellular surface as deduced from the complexity of GI1. This increased complexity as well as the presence of highly variable genes (e.g., major variable surface protein) might be advantageous for viral defence. If a virus initially binds to the protein other than the primary receptor, that would trap it in the initial phase of infection. This way, if several glycoproteins are exposed on the cellular surface, they would act as decoys, increasing the chance that the cell will survive the encounter with viruses.

In *Methanosarcina mazei*, it has been shown that the genes that lead to organismal growth in multicellular structures (located on a GI1 equivalent) are expressed only under certain environmental conditions [58, 59]. While the expression of *H. walsbyi* GI1 genes remains to be established, the presence of putative transcription regulators in the vicinity of these genes indicates that they might be expressed in response to a stimuli or environmental change. Such transient expression would in turn decrease the probability for virus to infect its host.

Recent research indicated that in haloarchaea N-glycosylation of proteins plays a role in predator–prey interplay: viral dependence on N-glycosylation machinery of the host results in strain-specific glycosylation of its proteins as recently demonstrated in *Halorubrum* sp. strain PV6 and haloarchaeal pleomorphic virus 1 [60]. Accordingly, changes in composition of the glycan that is added to the viral structural proteins diminished its ability to infect the cell [60]. From the genomic sequence of *H. walsbyi* HBSQ001 and C23 it is evident that the N-glycosylation of surface proteins, including the S-layer protein is probably strain-specific. Both genomic sequences have unique *agl* gene clusters and besides, the genes involved in synthesis of sialic acid are absent from the C23 genome [11, 35]. This difference could vary individual lineage’s susceptibility to viruses. Along these lines, some Archaea process surface proteins by distinct N-glycosylation pathways in responses to environmental changes, as recently showed in *Haloferax volcanii* [61].

## Conclusions

The present study demonstrates that fosmid library construction is a useful approach in population studies of species that are difficult to maintain under laboratory conditions. Screening of *H. walsbyi* enriched fosmid library yielded seven previously unknown versions of eHwalsbyiGI1. We showed that the diversity of eHwalsbyiGI1 arises through several mechanisms that include mobile genetic elements and homologous recombination. Detailed analysis of these regions showed that the *H. walsbyi* population in CR30 is composed of two major clades that differ in complexity of cell surface structures. While eHwalsbyiGI1 Clade I, represented by a single CR30 clone and the genome of isolate C23, contained completely syntenic sequences composed of only four genes, eHwalsbyiGI1 Clade II was complex, mosaic in structure, and contained conserved and variable genes. The latter included the gene that encodes the S-layer protein, a gene similar to halomucin that may contribute to capsule formation/multicellular growth and highly variable surface proteins that were preserved only in terms of synteny. The domain structure and the variability of these proteins led us to believe that their diversity arises as a consequence of trying to avoid predation by viruses. Sequence analysis indicated that *H. walsbyi* has several mechanisms that might provide viral defence including receptor masking, modifying the receptor proteins, and transient receptor expression.

## Methods

### Sequencing and assembly of genomic clones

The environmental genomic library used here was constructed as described in [27]. Fragments of metagenomic DNA extracted from the CR30 crystallizer of Santa Pola solar saltern and ranging in size from 35 to 40 Kb were cloned into pCC1Fos vector (Epicentre, USA) according to manufacturer’s instructions. The fosmids were replicated in *Escherichia coli* EPI300-T1^R^ cells. From this collection, the terminal sequences of 2948 inserts were determined by Sanger methodology generating approximately 2.4 Mb. This fosmid-end library was screened in order to identify clones whose end-sequences were significantly similar (BLASTN e_value ≤ 10^-5^) to either of the GI1 borders or to genes within the GI1. This way we identified eleven eHwalsbyi fosmids and isolated individual fosmid DNA using the QIAprep Spin Miniprep kit (QIAGEN, USA). The concentration of fosmid DNA was determined using Quant-iT™ PicoGreen ® dsDNA Reagent (Invitrogen, USA). Fosmid DNA was pyrosequenced (Roche 454 GS-FLX system by GATC, Konstanz, Germany) tagging each individual fosmid by using a multiplex identifier adaptor (MID). Sequencing revealed an average of 4500 reads per fosmid with average read length of 230 bp, meaning an average of 30 × coverage. Sequence assembly was performed using CLC Genomics Workbench 3 using a cut off of 97 % identity over 50 bp. All the fosmids, with the exception of eHwalsbyiGI1_5, were assembled in one single contig.

### Annotation of fosmids

Protein coding genes were predicted using the annotation package GLIMMER 3.02 [62] and were further manually curated. ORFs were compared to known proteins in the non-redundant GenBank database (http://www.ncbi.nlm.nih.gov/BLAST/) using BLASTX and ref_seq using DELTABLAST. All hits with an e-value greater than 10^-5^ were considered non-significant and ORFs containing less than 30 codons and without significant homology to other proteins were eliminated. To confirm the presence of domains in the detected CDS the Hmmpfam program of the HMMER package [63] was used. The hmm models for the protein domains were obtained from the Pfam database (http://pfam.sanger.ac.uk). The ORFs were further screened for domains using Interpro (http://www.ebi.ac.uk/interpro/). Transmembrane domains were predicted using TMHMM 2.0 [64]. t-RNAscan-SE 1.21 was used to predict any t-RNA in the sequences [65]. Putative signal peptide cleavage sites were predicted using SignalP, lipoproteins were predicted using LipoP, putative N- and O-glycosilation sites using NetNGlyc and NetOGlyc, subcellular localisation using TargetP and SecretomeP, all available from http://www.cbs.dtu.dk/services/.

### Sequence analysis

For comparative analyses, reciprocal BLASTN and TBLASTXs searches between the different fosmids were carried out, leading to the identification of regions of similarity. This way, four out of eleven fosmids were removed as falsely positive. To allow the interactive visualisation of genomic fragment comparisons between the complete genomes and eHwalsbyiGI1 sequences we used Artemis Comparison Tool ACTv.8 [66]. Alignment of genes annotated as major cell surface protein were generated using MAFFT available from (http://www.ebi.ac.uk/Tools/msa/mafft/) and were edited manually as necessary. This alignment was analysed by maximum likelihood (ML) in MEGA5 [67] by applying a Jones-Taylor-Thorton (JTT) model of sequence evolution and taking among-site variation into account using a four-category discrete approximation of a Γ distribution with a portion of invariable sites. GC content was identified using the ‘geecee’ program from EMBOSS package [68]. For tetranucleotide analysis, the tetranucleotide frequencies were computed using the ‘wordfreq’ program from the EMBOSS package [68], followed by principal component analysis in R package FactoMineR [69]. To calculate the Ka/Ks according to Liberles [70] we used Ka/Ks Calculator Tool (http://services.cbu.uib.no/tools/kaks). This service translates nucleotide sequences, calculates multiple sequence alignment, transforms it back to DNA and the resulting alignment is used to construct phylogenetic tree using least squares distance method of Jukes and Cantor. Then, at each site, the changes along the tree are counted to identify sites with an excess of nonsynonymous substitutions [71]. This analysis was with analysis performed using JCoDA, which accepts user-inputted unaligned or pre-aligned coding sequences, performs a codon-delimited alignment using ClustalW, and determines the dN/dS calculations using PAML (Phylogenetic Analysis Using Maximum Likelihood, yn00 and codeml) in order to identify regions and sites under evolutionary selection [48].

Fosmid sequences obtained and annotated in this study have been deposited in GenBank under the BioProject accession: PRJNA264323. The sequence of the *H. walsbyi* strain HBSQ001 is available under GenBank BioProject accession: PRJNA17185 and the *H. walsbyi* C23: PRJEA49335.

## Additional files

Additional file 1:
**Schematic representation of the strategy followed to select the eHwalsbyi fosmids clones from the environmental library FLAS CR30-2002.** Sequences that hit inside or in the neighboring areas of the GI1 of the *H. walsbyi* HSBQ001 strain genome were selected. Colored arrows indicate selected fosmid-ends and are placed accordingly to their similarity (y axes) and matching position to the reference sequence (x axes). Information of each corresponding pair-end is indicated, the position in the HBSQ001 genome and GC content. (PPTX 76 kb) Additional file 2:
**Functional assignment of the ORFs contained in the eHwalsbyiGI1s fragments using the COG database: (A) RNA processing and modification; (C) Energy production and conversion; (G) Carbohydrate transport and metabolism; (N) Cell motility; (U) Intracellular trafficking, secretion, and vesicular transport; (E) Amino acid transport and metabolism; (F) Nucleotide transport and metabolism; (T) Signal transduction mechanisms; (K) Transcription; (M) Cell wall/membrane biogenesis; (P) Inorganic ion transport and metabolism; (S) Function unknown; (L) Replication, recombination and repair; (R) General function prediction only.** (PPTX 66 kb) Additional file 3:
**Distribution patterns of COG-associated proteins.** (DOCX 27 kb) Additional file 4:
**Annotation details for ORFs detected in this study.** (DOCX 66 kb) Additional file 5:
**Average frequencies of individual amino acids in ORFs found in the eHwalsbyiGI1s studied here, eHwalsbyi559 [** 10 **] (blue), and genomes of** ***H. walsbyi*** **HBSQ001 and C23 (red)**. (PPTX 49 kb) Additional file 6:
**Localisation, length and consensus sequence of repeats detected in this study using XSTREAM** (DOCX 31 kb) Additional file 7:
**List of putative recombination events as detected by RDP software.** (DOCX 18 kb) Additional file 8:
**Ka/Ks values for most frequent groups of gene homologs detected in this study.** (DOCX 25 kb)
