# Supplementary material for: Diversity of the cell-wall associated genomic island of the archaeon Haloquadratum walsbyi
Source: BMC Genomics. 2015 Aug 13;16(1):603. doi: 10.1186/s12864-015-1794-8 (PMC4535781; doi:10.1186/s12864-015-1794-8)
Supplement: Additional file 1: — Schematic representation of the strategy followed to select the eHwalsbyi fosmids clones from the environmental library FLAS CR30-2002. Sequences that hit inside or in the neighboring areas of the GI1 of the H. walsbyi HSBQ001 strain genome were selected. Colored arrows indicate selected fosmid-ends and are placed accordingly to their similarity (y axes) and matching position to the reference sequence (x axes). Information of each corresponding pair-end is indicated, the position in the HBSQ001 genome and GC content. (PPTX 76 kb) [file 12864_2015_1794_MOESM1_ESM.pptx]

## Slide 1
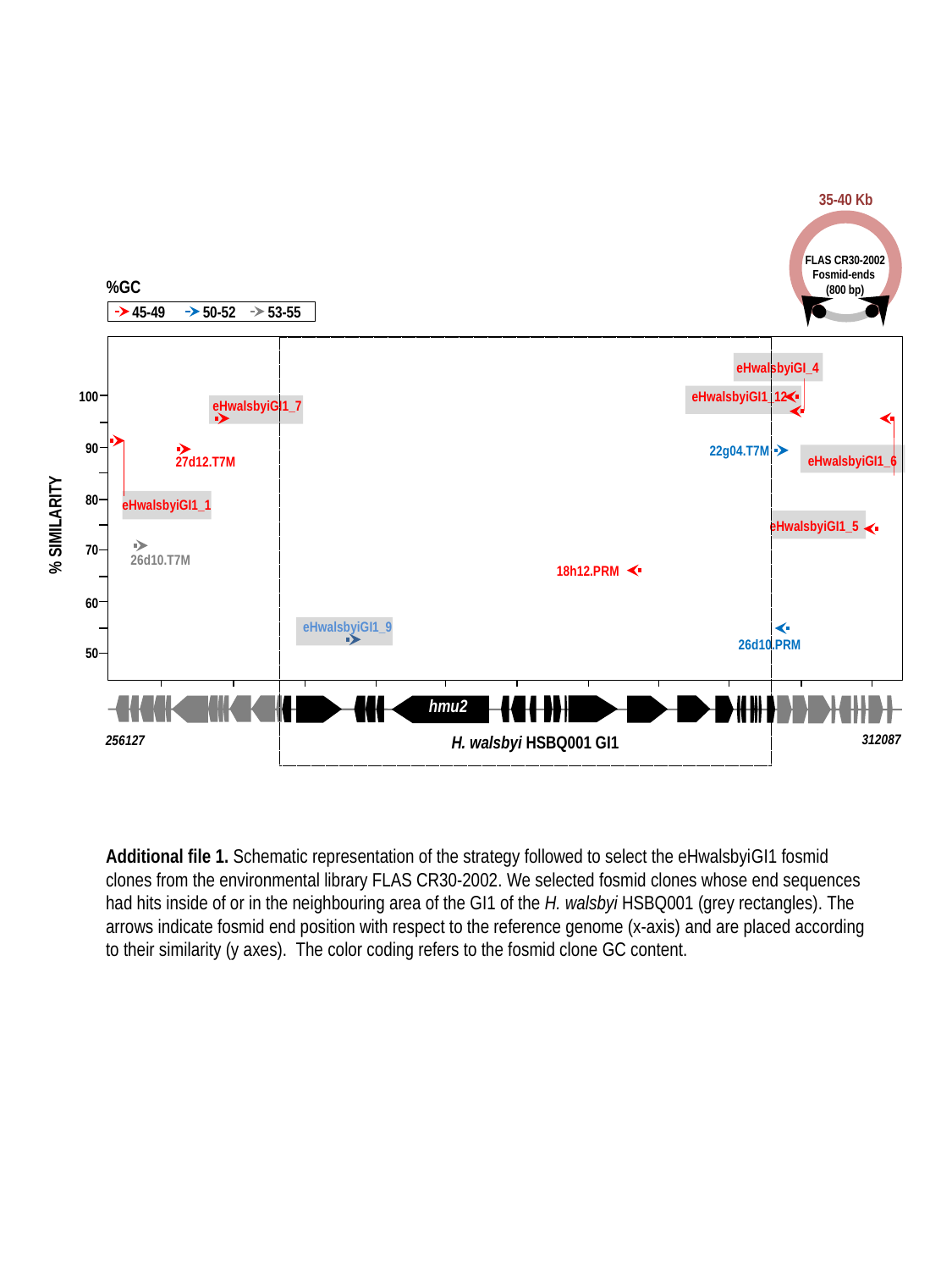

35-40 Kb
FLAS CR30-2002
Fosmid-ends
(800 bp)
%GC
45-49
50-52
53-55
eHwalsbyiGI_4
eHwalsbyiGI1_12
100
eHwalsbyiGI1_7
90
22g04.T7M
eHwalsbyiGI1_6
27d12.T7M
80
eHwalsbyiGI1_1
eHwalsbyiGI1_5
% SIMILARITY
70
26d10.T7M
18h12.PRM
60
eHwalsbyiGI1_9
26d10.PRM
50
hmu2
312087
H. walsbyi HSBQ001 GI1
256127
Additional file 1. Schematic representation of the strategy followed to select the eHwalsbyiGI1 fosmid clones from the environmental library FLAS CR30-2002. We selected fosmid clones whose end sequences had hits inside of or in the neighbouring area of the GI1 of the H. walsbyi HSBQ001 (grey rectangles). The arrows indicate fosmid end position with respect to the reference genome (x-axis) and are placed according to their similarity (y axes). The color coding refers to the fosmid clone GC content.
