# Supplementary material for: Diversity of the cell-wall associated genomic island of the archaeon Haloquadratum walsbyi
Source: BMC Genomics. 2015 Aug 13;16(1):603. doi: 10.1186/s12864-015-1794-8 (PMC4535781; doi:10.1186/s12864-015-1794-8)
Supplement: Additional file 2: — Functional assignment of the ORFs contained in the eHwalsbyiGI1s fragments using the COG database: (A) RNA processing and modification; (C) Energy production and conversion; (G) Carbohydrate transport and metabolism; (N) Cell motility; (U) Intracellular trafficking, secretion, and vesicular transport; (E) Amino acid transport and metabolism; (F) Nucleotide transport and metabolism; (T) Signal transduction mechanisms; (K) Transcription; (M) Cell wall/membrane biogenesis; (P) Inorganic ion transport and metabolism; (S) Function unknown; (L) Replication, recombination and repair; (R) General function prediction only. (PPTX 66 kb) [file 12864_2015_1794_MOESM2_ESM.pptx]

## Slide 1
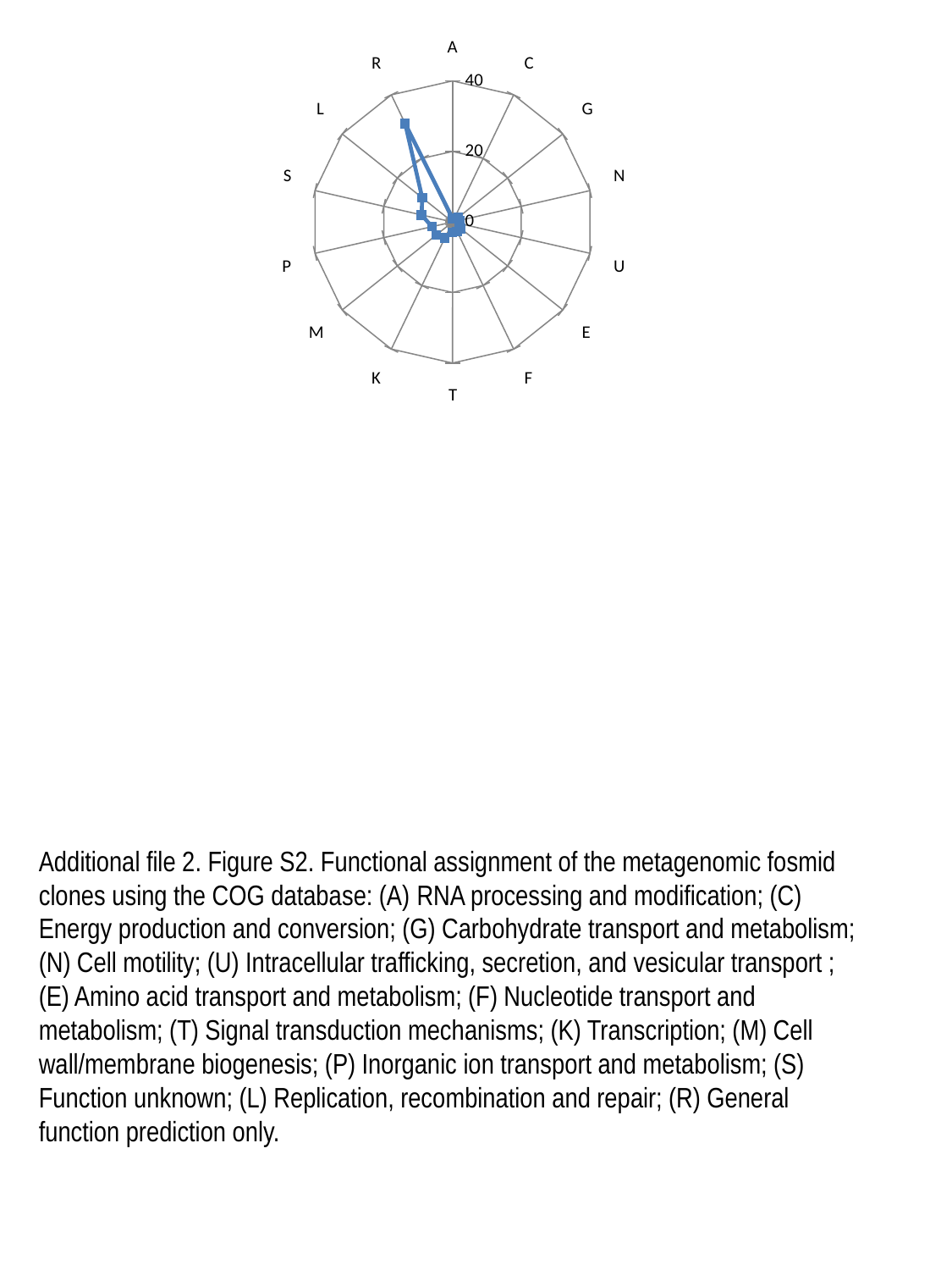

### Chart
| Category | |
|---|---|
| A | 1.0 |
| C | 1.0 |
| G | 2.0 |
| N | 2.0 |
| U | 2.0 |
| E | 3.0 |
| F | 3.0 |
| T | 3.0 |
| K | 5.0 |
| M | 6.0 |
| P | 6.0 |
| S | 9.0 |
| L | 11.0 |
| R | 31.0 |Additional file 2. Figure S2. Functional assignment of the metagenomic fosmid clones using the COG database: (A) RNA processing and modification; (C) Energy production and conversion; (G) Carbohydrate transport and metabolism; (N) Cell motility; (U) Intracellular trafficking, secretion, and vesicular transport ; (E) Amino acid transport and metabolism; (F) Nucleotide transport and metabolism; (T) Signal transduction mechanisms; (K) Transcription; (M) Cell wall/membrane biogenesis; (P) Inorganic ion transport and metabolism; (S) Function unknown; (L) Replication, recombination and repair; (R) General function prediction only.
