# Supplementary material for: Diversity of the cell-wall associated genomic island of the archaeon Haloquadratum walsbyi
Source: BMC Genomics. 2015 Aug 13;16(1):603. doi: 10.1186/s12864-015-1794-8 (PMC4535781; doi:10.1186/s12864-015-1794-8)
Supplement: Additional file 3: — Distribution patterns of COG-associated proteins. (DOCX 27 kb) [file 12864_2015_1794_MOESM3_ESM.docx]

# Additional file 3. Distribution patterns of COG-assigned proteins.

| eHwalsbyiGI1 query | Hit | E-value | Description | class | class description |
| --- | --- | --- | --- | --- | --- |
| 1_CDS39 | COG0589 | 5,00E-12 | Universal stress protein UspA  and related nucleotide-binding proteins | T | Signal transduction  mechanisms |
| 1_CDS31 | COG3889 | 1,00E-10 | Predicted solute binding protein | R | General function  prediction only |
| 1_CDS30 | COG3889 | 2,00E-10 | Predicted solute binding protein | R | General function  prediction only |
| 1_CDS35 | COG1361 | 5,00E-27 | S-layer domain | M | Cell wall/membrane  /envelope biogenesis |
| 1_CDS41 | COG3379 | 1,00E-112 | Uncharacterized conserved protein | S | Function unknown |
| 1_CDS33 | COG1309 | 7,00E-08 | Transcriptional regulator | K | Transcription |
| 1_CDS12 | COG0451 | 6,00E-17 | Nucleoside-diphosphate-sugar  epimerases | MG | Multiple classes |
| 1_CDS45 | COG1387 | 3,00E-21 | Histidinol phosphatase and related  hydrolases of the PHP family | ER | Multiple classes |
| 1_CDS02 | COG0675 | 2,00E-24 | Transposase and inactivated derivatives | L | Replication, recombination  and repair |
| 1_CDS26 | COG0630 | 8,00E-32 | Type IV secretory pathway, VirB11  components, and related ATPases  involved in archaeal flagella biosynthesis | NU | Multiple classes |
| 1_CDS10 | COG0667 | 3,00E-29 | Predicted oxidoreductases (related  to aryl-alcohol dehydrogenases) | C | Energy production  and conversion |
| 1_CDS44 | COG1796 | 2,00E-72 | DNA polymerase IV (family X) | L | Replication, recombination  and repair |
| 1_CDS44 | COG1387 | 0.002 | Histidinol phosphatase and  related hydrolases of the PHP family | ER | Multiple classes |
| 1_CDS38 | COG1033 | 1,00E-138 | Predicted exporters of the  RND superfamily | R | General function  prediction only |
| 1_CDS46 | COG1656 | 2,00E-21 | Uncharacterized conserved protein | S | Function unknown |
| 1_CDS04 | COG0494 | 1,00E-05 | NTP pyrophosphohydrolases  including oxidative damage repair enzymes | LR | Multiple classes |
| 1_CDS42 | COG3462 | 1,00E-06 | Predicted membrane protein | S | Function unknown |
| 4_CDS44 | COG1033 | 3,00E-67 | Predicted exporters of the RND superfamily | R | General function  prediction only |
| 4_CDS19 | COG3889 | 4,00E-08 | Predicted solute binding protein | R | General function  prediction only |
| 4_CDS38 | COG3889 | 3,00E-07 | Predicted solute binding protein | R | General function  prediction only |
| 4_CDS35 | COG3889 | 6,00E-04 | Predicted solute binding protein | R | General function  prediction only |
| 4_CDS41 | COG1309 | 9,00E-09 | Transcriptional regulator | K | Transcription |
| 4_CDS6 | COG3889 | 8,00E-07 | Predicted solute binding protein | R | General function  prediction only |
| 4_CDS13 | COG1572 | 0.001 | Uncharacterized conserved protein | S | Function unknown |
| 4_CDS1 | COG3889 | 2,00E-10 | Predicted solute binding protein | R | General function  prediction only |
| 4_CDS8 | COG3889 | 4,00E-08 | Predicted solute binding protein | R | General function  prediction only |
| 4_CDS8 | COG4907 | 0.009 | Predicted membrane protein | S | Function unknown |
| 4_CDS4 | COG5183 | 0.004 | Protein involved in mRNA  turnover and stability | A | RNA processing  and modification |
| 4_CDS4 | COG3889 | 0.007 | Predicted solute binding protein | R | General function  prediction only |
| 4_CDS43 | COG1361 | 3,00E-29 | S-layer domain | M | Cell wall/membrane/  envelope biogenesis |
| 4_CDS32 | COG0614 | 4,00E-25 | ABC-type Fe3+-hydroxamate  transport system, periplasmic component | P | Inorganic ion transport  and metabolism |
| 5_CDS12 | COG3889 | 4,00E-06 | Predicted solute binding protein | R | General function  prediction only |
| 5_CDS11 | COG0737 | 2,00E-49 | 5'-nucleotidase/2',3'-cyclic  phosphodiesterase and related esterases | F | Nucleotide transport  and metabolism |
| 5_CDS14 | COG3889 | 1,00E-09 | Predicted solute binding protein | R | General function  prediction only |
| 5_CDS7 | COG0675 | 9,00E-10 | Transposase and inactivated  derivatives | L | Replication,  recombination and repair |
| 5_CDS17 | COG1361 | 8,00E-29 | S-layer domain | M | Cell wall/membrane/  envelope biogenesis |
| 5_gene_1 | COG3889 | 8,00E-10 | Predicted solute binding protein | R | General function  prediction only |
| 5_CDS18 | COG1033 | 1,00E-121 | Predicted exporters of the  RND superfamily | R | General function  prediction only |
| 5_CDS9 | COG0614 | 2,00E-24 | ABC-type Fe3+-hydroxamate  transport system, periplasmic  component | P | Inorganic ion transport  and metabolism |
| 5_CDS3 | COG1430 | 3,00E-18 | Uncharacterized conserved protein | S | Function unknown |
| 5_CDS16 | COG1309 | 9,00E-05 | Transcriptional regulator | K | Transcription |
| 6_CDS29 | COG1361 | 1,00E-28 | S-layer domain | M | Cell wall/membrane/  envelope biogenesis |
| 6_CDS27 | COG1309 | 9,00E-09 | Transcriptional regulator | K | Transcription |
| 6_CDS34 | COG3379 | 1,00E-112 | Uncharacterized conserved protein | S | Function unknown |
| 6_CDS42 | COG5422 | 0.001 | RhoGEF, Guanine nucleotide  exchange factor for  Rho/Rac/Cdc42-like GTPases | T | Signal transduction  mechanisms |
| 6_CDS42 | COG1263 | 0.008 | Phosphotransferase system  IIC components,  glucose/maltose/N-acetylglucosamine-specific | G | Carbohydrate transport  and metabolism |
| 6_CDS38 | COG1796 | 2,00E-72 | DNA polymerase IV (family X) | L | Replication, recombination  and repair |
| 6_CDS38 | COG1387 | 2,00E-42 | Histidinol phosphatase and  related hydrolases of the PHP family | ER | Multiple classes |
| 6_CDS33 | COG0589 | 8,00E-12 | Universal stress protein UspA  and related nucleotide-binding proteins | T | Signal transduction  mechanisms |
| 6_CDS1 | COG3889 | 2,00E-05 | Predicted solute binding protein | R | General function  prediction only |
| 6_CDS23 | COG3889 | 1,00E-07 | Predicted solute binding protein | R | General function  prediction only |
| 6_CDS32 | COG1033 | 1,00E-138 | Predicted exporters of the  RND superfamily | R | General function  prediction only |
| 6_CDS35 | COG3462 | 4,00E-07 | Predicted membrane protein | S | Function unknown |
| 6_CDS2 | COG3889 | 2,00E-06 | Predicted solute binding protein | R | General function  prediction only |
| 6_CDS20 | COG0614 | 7,00E-24 | ABC-type Fe3+-hydroxamate  transport system, periplasmic component | P | Inorganic ion transport  and metabolism |
| 6_CDS40 | COG1656 | 9,00E-22 | Uncharacterized conserved protein | S | Function unknown |
| 7_CDS5 | COG3889 | 2,00E-11 | Predicted solute binding protein | R | General function  prediction only |
| 7_CDS9 | COG3889 | 1,00E-06 | Predicted solute binding protein | R | General function  prediction only |
| 7_CDS12 | COG3889 | 3,00E-07 | Predicted solute binding protein | R | General function  prediction only |
| 7_CDS6 | COG3039 | 6,00E-30 | Transposase and inactivated  derivatives, IS5 family | L | Replication, recombination  and repair |
| 7_CDS2 | COG0630 | 2,00E-32 | Type IV secretory pathway,  VirB11 components, and related  ATPases involved in archaeal  flagella biosynthesis | NU | Multiple classes |
| 7_CDS16 | COG0614 | 1,00E-23 | ABC-type Fe3+-hydroxamate  transport system, periplasmic  component | P | Inorganic ion transport  and metabolism |
| 9_CDS36 | COG3415 | 3,00E-15 | Transposase and inactivated  derivatives | L | Replication, recombination  and repair |
| 9_CDS36 | COG3335 | 2,00E-04 | Transposase and inactivated  derivatives | L | Replication, recombination  and repair |
| 9_CDS33 | COG0675 | 3,00E-05 | Transposase and inactivated  derivatives | L | Replication, recombination  and repair |
| 9_CDS52 | COG0614 | 5,00E-25 | ABC-type Fe3+-hydroxamate t  ransport system, periplasmic  component | P | Inorganic ion transport  and metabolism |
| 9_CDS3 | COG3889 | 7,00E-07 | Predicted solute binding protein | R | General function  prediction only |
| 9_CDS26 | COG3889 | 6,00E-08 | Predicted solute binding protein | R | General function  prediction only |
| 12_CDS21 | COG0675 | 7,00E-18 | Transposase and inactivated  derivatives | L | Replication, recombination  and repair |
| 12_CDS31 | COG0737 | 6,00E-25 | 5'-nucleotidase/2',3'-cyclic  Phosphodiesterase  and related esterases | F | Nucleotide transport  and metabolism |
| 12_CDS30 | COG0737 | 4,00E-20 | 5'-nucleotidase/2',3'-cyclic  phosphodiesterase  and related esterases | F | Nucleotide transport  and metabolism |
| 12_CDS34 | COG3889 | 9,00E-10 | Predicted solute binding protein | R | General function  prediction only |
| 12_CDS40 | COG1033 | 2,00E-71 | Predicted exporters  of the RND superfamily | R | General function  prediction only |
| 12_CDS37 | COG1309 | 8,00E-09 | Transcriptional regulator | K | Transcription |
| 12_CDS10 | COG3039 | 2,00E-19 | Transposase and  inactivated derivatives, IS5 family | L | Replication, recombination  and repair |
| 12_CDS32 | COG3889 | 4,00E-06 | Predicted solute binding protein | R | General function  prediction only |
| 12_CDS28 | COG0614 | 2,00E-24 | ABC-type Fe3+-hydroxamate  transport system,  periplasmic component | P | Inorganic ion transport  and metabolism |
| 12_CDS39 | COG1361 | 4,00E-29 | S-layer domain | M | Cell wall/membrane/  envelope biogenesis |
