# Supplementary material for: Diversity of the cell-wall associated genomic island of the archaeon Haloquadratum walsbyi
Source: BMC Genomics. 2015 Aug 13;16(1):603. doi: 10.1186/s12864-015-1794-8 (PMC4535781; doi:10.1186/s12864-015-1794-8)
Supplement: Additional file 4: — Annotation details for ORFs detected in this study. (DOCX 66 kb) [file 12864_2015_1794_MOESM4_ESM.docx]

Additional file 4. Annotation details for ORFs detected in this study.

| CDS | start | end | annotation | best deltablast hit | identity (%) | best hit accession number | SignalP | TatP (No Tat motif) | Number of predicted THMs | TargetP (S-secreted) | Localisation | Cleavage site | NetOGlyc | NetNGlyc |
| --- | --- | --- | --- | --- | --- | --- | --- | --- | --- | --- | --- | --- | --- | --- |
| 1_CDS02 | 2 | 875 | IS1341-type transposase | Hqrw_1219 IS1341-type transposase Haloquadratum walsbyi C23 | 99% | [WP_014555099.1](http://www.ncbi.nlm.nih.gov/protein/504367997?report=genbank&log$=prottop&blast_rank=1&RID=W7RDJ8DV014) | NO |  | 0 |  | CYT |  | YES | NO |
| 1_CDS04 | 3 | 1635 | nudix family protein | HQ1178A nudix family protein [ *Haloquadratum walsbyi DSM 16790* ] | 99% | [WP_011570473.1](http://www.ncbi.nlm.nih.gov/protein/499889739?report=genbank&log$=prottop&blast_rank=1&RID=W7RDXDD601R) | NO |  | 0 |  | CYT |  | YES | NO |
| 1_CDS05 | 7 | 2442 | hypothetical protein | Hqrw_1221 hypothetical protein [ *Haloquadratum walsbyi C23* ] | 99% | [WP_014555101.1](http://www.ncbi.nlm.nih.gov/protein/504367999?report=genbank&log$=prottop&blast_rank=1&RID=W7RE7K4P01R) | NO |  | 0 |  | CYT |  | YES | NO |
| 1_CDS06 | 5 | 3773 | glycosyltransferase-like protein | glycosyl transferase family 2 [Haloquadratum walsbyi] | 99% | [WP_011570475.1](http://www.ncbi.nlm.nih.gov/protein/499889741?report=genbank&log$=prottop&blast_rank=1&RID=W7REJBNX01R) | NO |  | 0 |  | CYT |  | YES | NO |
| 1_CDS09 | 11 | 4781 | hypothetical protein | N/A |  |  | NO |  | 0 |  | CYT |  | NO | NO |
| 1_CDS10 | 5 | 5955 | aldo/keto reductase family protein | HQ1182A aldo/keto reductase [ *Haloquadratum walsbyi DSM 16790* ] | 99% | [WP_011570476.1](http://www.ncbi.nlm.nih.gov/protein/499889742?report=genbank&log$=prottop&blast_rank=1&RID=W7S45DSW01R) | NO |  | 0 |  | CYT |  | YES | NO |
| 1_CDS12 | 1 | 7109 | sugar epimerase | HQ1183A sugar epimerase/dehydratase-like protein [ *Haloquadratum walsbyi DSM 16790* ] | 98% | [WP_011570477.1](http://www.ncbi.nlm.nih.gov/protein/499889743?report=genbank&log$=prottop&blast_rank=1&RID=W7SFYDMS01R) | NO |  | 0 |  | CYT |  | YES | NO |
| 1_CDS13 | 50 | 7573 | hypothetical protein | HQ1184A hypothetical protein [ *Haloquadratum walsbyi DSM 16790* ] | 100% | [WP_011570478.1](http://www.ncbi.nlm.nih.gov/protein/499889744?report=genbank&log$=prottop&blast_rank=1&RID=W7S4UJHF01R) | NO |  | 0 |  | CYT |  | YES | NO |
| 1_CDS14 | 7 | 10336 | hypothetical protein | HQ1185A hypothetical protein [ *Haloquadratum walsbyi DSM 16790* ] | 99% | [WP_011570479.1](http://www.ncbi.nlm.nih.gov/protein/499889745?report=genbank&log$=prottop&blast_rank=1&RID=W7S5JFY101R) | NO |  | 0 |  | CYT |  | YES | NO |
| 1_CDS15 | 21 | 10818 | hypothetical protein | HQ1185A hypothetical protein [ *Haloquadratum walsbyi DSM 16790* ] | 99% | [WP_011570479.1](http://www.ncbi.nlm.nih.gov/protein/499889745?report=genbank&log$=prottop&blast_rank=1&RID=W7S5JFY101R) | NO |  | 1 | S | CYT |  | YES | NO |
| 1_CDS17 | 7 | 11675 | hypothetical protein | Hqrw_1229 hypothetical protein [ *Haloquadratum walsbyi C23* ] | 99% | [WP_014555107.1](http://www.ncbi.nlm.nih.gov/protein/504368005?report=genbank&log$=prottop&blast_rank=1&RID=W7SP5ZDE01R) | NO |  | 1 | S | SpI | cleavage=21-22 | YES | YES |
| 1_CDS18 | 14 | 12106 | hypothetical protein | HQ1187A hypothetical protein [ Haloquadratum walsbyi DSM 16790 ] | 96% | [WP_011570481.1](http://www.ncbi.nlm.nih.gov/protein/499889747?report=genbank&log$=prottop&blast_rank=1&RID=W7SPGANG01R) | NO |  | 1 |  | CYT |  | YES | NO |
| 1_CDS20 | 17 | 12644 | hypothetical protein | HQ1188A hypothetical protein [ Haloquadratum walsbyi DSM 16790 ] | 99% | [WP_014555109.1](http://www.ncbi.nlm.nih.gov/protein/504368007?report=genbank&log$=prottop&blast_rank=1&RID=W7SR6C3R014) | NO |  | 1 | S | TMH |  | NO | YES |
| 1_CDS22 | 1 | 13108 | hypothetical protein | Hqrw_1232 type II/IV secretion system transmembrane protein [ *Haloquadratum walsbyi C23* ] | 98% | [WP_014555110.1](http://www.ncbi.nlm.nih.gov/protein/504368008?report=genbank&log$=prottop&blast_rank=1&RID=W7SRF060015) | NO |  | 2 |  | CYT |  | YES | NO |
| 1_CDS24 | 48 | 14640 | hypothetical protein | HQ1189A hypothetical protein [ *Haloquadratum walsbyi DSM 16790* ] | 98% | [WP_011570483.1](http://www.ncbi.nlm.nih.gov/protein/499889749?report=genbank&log$=prottop&blast_rank=1&RID=WA4H6HJG01R) | 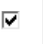   \| NO \| \| --- \| |  | 6 |  | CYT |  | NO | NO |
| 1_CDS26 | 124 | 16724 | type II IV secretion system proteins VirB11/TadA (ATPase) | Hqrw_1233 type II/IV secretion system ATPase protein [ *Haloquadratum walsbyi C23* ] | 99% | [WP_014555111.1](http://www.ncbi.nlm.nih.gov/protein/504368009?report=genbank&log$=prottop&blast_rank=1&RID=WA4T5PJY01R) | NO |  | 0 |  | CYT |  | YES | NO |
| 1_CDS27 | 26 | 17282 | hypothetical protein | Hqrw_1234 hypothetical protein [ *Haloquadratum walsbyi C23* ] | 96% | [WP_014555112.1](http://www.ncbi.nlm.nih.gov/protein/504368010?report=genbank&log$=prottop&blast_rank=1&RID=WA4JJ3TB015) | YES |  | 1 | S | SpI | cleavage=18-19 | YES | NO |
| 1_CDS29 | 4 | 18142 | dentin sialophosphoprotein precursor | Hqrw_1233 type II/IV secretion system ATPase protein [ *Haloquadratum walsbyi C23* ] | 99% | [WP_014555111.1](http://www.ncbi.nlm.nih.gov/protein/504368009?report=genbank&log$=prottop&blast_rank=1&RID=WA4T5PJY01R) | NO |  | 0 |  | CYT |  | YES | NO |
| 1_CDS30 | 2 | 22185 | cell surface glycoprotein | Hqrw_1236 probable cell surface glycoprotein [ *Haloquadratum walsbyi C23* ] | 91% | [WP_014555114.1](http://www.ncbi.nlm.nih.gov/protein/504368012?report=genbank&log$=prottop&blast_rank=1&RID=WA4KJ2CE014) | NO |  | 2 | S | TMH |  | YES | NO |
| 1_CDS31 | 7 | 24901 | S-layer protein | S-layer glycoprotein [ *Haloquadratum walsbyi C23* ] | 97% | [WP_014555115.1](http://www.ncbi.nlm.nih.gov/protein/504368013?report=genbank&log$=prottop&blast_rank=1&RID=WA4MEYDZ015) | NO |  | 2 | S | SpI | cleavage=31-32 | YES | NO |
| 1_CDS32 | 1 | 25808 | hypothetical protein | hypothetical protein [Haloquadratum walsbyi] | 95% | [WP_021049929.1](http://www.ncbi.nlm.nih.gov/protein/544611578?report=genbank&log$=prottop&blast_rank=1&RID=WA4NJ3XT015) | NO |  | 4 | S | TMH |  | NO | NO |
| 1_CDS33 | 4 | 26663 | transcription regulator-like protein | Hqrw_1239 TetR family transcription regulator [ *Haloquadratum walsbyi C23* ] | 98% | [WP_014555117.1](http://www.ncbi.nlm.nih.gov/protein/504368015?report=genbank&log$=prottop&blast_rank=1&RID=WA53S765014) | NO |  | 0 |  | CYT |  | YES | NO |
| 1_CDS35 | 1 | 28428 | S-layer domain protein | HQ1214A hypothetical protein [ *Haloquadratum walsbyi DSM 16790* ] | 95% | [WP_011570505.1](http://www.ncbi.nlm.nih.gov/protein/499889771?report=genbank&log$=prottop&blast_rank=1&RID=WA541669014) | YES |  | 2 | S | SpI | cleavage=25-26 | YES | YES |
| 1_CDS38 | 1 | 30766 | RND superfamily multidrug efflux system protein | Hqrw_1241 RND superfamily permease [ *Haloquadratum walsbyi C23* ] | 98% | [WP_011570506.1](http://www.ncbi.nlm.nih.gov/protein/499889772?report=genbank&log$=prottop&blast_rank=1&RID=WA54BS8D015) | NO |  | 13 | S | SpI | cleavage=37-38 | YES | YES |
| 1_CDS39 | 223 | 31256 | stress response protein | Hqrw_1242 UspA domain protein [ *Haloquadratum walsbyi C23* ] | 99% | [WP_014555120.1](http://www.ncbi.nlm.nih.gov/protein/504368018?report=genbank&log$=prottop&blast_rank=1&RID=WA54NA9Y014) | NO |  | 0 |  | CYT |  | NO | NO |
| 1_CDS41 | 3 | 32765 | hypothetical protein | Hqrw_1243 probable phosphodiesterase [ *Haloquadratum walsbyi C23* ] | 99% | [WP_014555121.1](http://www.ncbi.nlm.nih.gov/protein/504368019?report=genbank&log$=prottop&blast_rank=1&RID=WA550HJ001R) | NO |  | 0 |  | CYT |  | NO | NO |
| 1_CDS42 | 455 | 33548 | hypothetical protein | Hqrw_1244 DUF2078 family protein [ *Haloquadratum walsbyi C23* ] | 98% | [WP_014555122.1](http://www.ncbi.nlm.nih.gov/protein/504368020?report=genbank&log$=prottop&blast_rank=1&RID=WA55B8NW01R) | NO |  | 2 | S | TMH |  | YES | YES |
| 1_CDS43 | 11 | 34174 | hypothetical protein | HQ1219A hypothetical protein [ Haloquadratum walsbyi DSM 16790 ] | 100% | [WP_011570510.1](http://www.ncbi.nlm.nih.gov/protein/499889776?report=genbank&log$=prottop&blast_rank=1&RID=WA5PG83X01R) | NO |  | 0 |  | CYT |  | YES | NO |
| 1_CDS44 | 1 | 35622 | family X DNA-dependent DNA polymerase | hypothetical protein [Bacillus subtilis] | 28% | [WP_029318208.1](http://www.ncbi.nlm.nih.gov/protein/657203776?report=genbank&log$=prottop&blast_rank=1&RID=WA5PT45201R) | NO |  | 0 |  | CYT |  | YES | YES |
| 1_CDS45 | 106 | 35960 | family X DNA-dependent DNA polymerase | Moth_1748 PHP-like protein [ *Moorella thermoacetica ATCC 39073* ] | 61% | [WP_004057527.1](http://www.ncbi.nlm.nih.gov/protein/490158862?report=genbank&log$=prottop&blast_rank=5&RID=WA5R9MGZ01R) | NO |  | 0 |  | CYT |  | NO | NO |
| 1_CDS46 | 1 | 36679 | hypothetical protein | HQ1221A hypothetical protein [ *Haloquadratum walsbyi DSM 16790* ] | 98% | [WP_011570512.1](http://www.ncbi.nlm.nih.gov/protein/499889778?report=genbank&log$=prottop&blast_rank=1&RID=WA5RPNBY01R) | NO |  | 0 |  | CYT |  | YES | NO |
| 4_CDS1 | 3 | 3318 | cell surface glycoprotein | HQ1193A cell surface glycoprotein [ Haloquadratum walsbyi DSM 16790 ] | 98% | [WP_011570487.1](http://www.ncbi.nlm.nih.gov/protein/499889753?report=genbank&log$=prottop&blast_rank=1&RID=WCDKSFB201N) | NO |  | 1 |  | CYT |  | YES | NO |
| 4_CDS2 | 4 | 3910 | hypothetical protein | transition state regulator Abh [Bacillus cereus] | 43% | [WP_002035310.1](http://www.ncbi.nlm.nih.gov/protein/487962020?report=genbank&log$=prottop&blast_rank=1&RID=WCDPGGN8013) | NO |  | 0 |  | CYT |  | NO | NO |
| 4_CDS4 | 171 | 5839 | cell surface protein | hypothetical protein [uncultured haloarchaeon eHwalsbyi559 | 95% | [ABQ76147.1](http://www.ncbi.nlm.nih.gov/protein/148508365?report=genbank&log$=prottop&blast_rank=1&RID=WCDW1R75013) | YES |  | 1 | S | SpI | cleavage=21-22 | YES | NO |
| 4_CDS6 | 19 | 6598 | cell surface glycoprotein | HQ1196A cell surface glycoprotein [ *Haloquadratum walsbyi DSM 16790* ] | 100% | [WP_011570490.1](http://www.ncbi.nlm.nih.gov/protein/499889756?report=genbank&log$=prottop&blast_rank=1&RID=WCDW91HE016) | YES |  | 0 | S | SpII | cleavage=20-21 | YES | NO |
| 4_CDS7 | 3 | 7016 | hypothetical protein | N/A |  |  | NO |  | 0 |  | CYT |  | NO | YES |
| 4_CDS8 | 234 | 8010 | probable cell surface glycoprotein | MmTuc01_3020 hypothetical protein [ *Methanosarcina mazei Tuc01* ] | 22% | [WP_015412926.1](http://www.ncbi.nlm.nih.gov/protein/505225824?report=genbank&log$=prottop&blast_rank=1&RID=WCDX2Z93016) | NO | YES | 1 |  | CYT |  | YES | NO |
| 4_CDS13 | 67 | 14515 | halomucin2 | hmu2 cell surface glycoprotein [ *Haloquadratum walsbyi DSM 16790* ] | 98% | [WP_011570491.1](http://www.ncbi.nlm.nih.gov/protein/499889757?report=genbank&log$=prottop&blast_rank=1&RID=WCH4850Z013) | NO |  | 0 |  | CYT |  | NO | YES |
| 4_CDS11 | 120 | 15217 | cell surface glycoprotein | hypothetical protein NBRGN_068_00020 [Nocardia brasiliensis NBRC 14402] | 32% | [GAJ84011.1](http://www.ncbi.nlm.nih.gov/protein/635329474?report=genbank&log$=prottop&blast_rank=1&RID=WCDYDX2D01N) | NO |  | 0 |  | CYT |  | YES | YES |
| 4_CDS15 | 23 | 17187 | cell surface glycoprotein, carbohydrate-binding protein | hmu2 cell surface glycoprotein [ *Haloquadratum walsbyi DSM 16790* ] | 41% | [WP_011570491.1](http://www.ncbi.nlm.nih.gov/protein/499889757?report=genbank&log$=prottop&blast_rank=1&RID=WCH2BW7301N) | NO |  | 1 | S | SpI | cleavage=38-39 | YES | NO |
| 4_CDS18 | 25 | 18342 | hypothetical protein | hypothetical protein HQ_1199A [Haloquadratum walsbyi DSM 16790] | 97% | [CAJ51328.2](http://www.ncbi.nlm.nih.gov/protein/403212799?report=genbank&log$=prottop&blast_rank=1&RID=WCH2UA8601N) | NO |  | 0 |  | CYT |  | YES | NO |
| 4_CDS19 | 18455 | 20692 | subtilisin-like serine protease | subtilisin-like serine protease [uncultured haloarchaeon] | 95% | [ABQ76140.1](http://www.ncbi.nlm.nih.gov/protein/148508358?report=genbank&log$=prottop&blast_rank=1&RID=WCH37JWG01N) | NO |  | 1 | S | SpI | cleavage=28-29 | YES | YES |
| 4_CDS22 | 21328 | 21453 | hypothetical protein | N/A |  |  | NO |  | 0 |  | CYT |  | NO | NO |
| 4_CDS23 | 21456 | 22016 | hypothetical protein | hypothetical protein (uncultured haloarchaeon eHwalsbyi559) | 97% | [ABQ76139.1](http://www.ncbi.nlm.nih.gov/protein/148508357?report=genbank&log$=prottop&blast_rank=1&RID=WTRR2R1U014) | NO |  | 0 | S | CYT |  | YES | YES |
| 4_CDS25 | 22398 | 22604 | putative transcription regulator | hypothetical protein (uncultured haloarchaeon eHwalsbyi559) | 99% | [ABQ76138.1](http://www.ncbi.nlm.nih.gov/protein/148508356?report=genbank&log$=prottop&blast_rank=1&RID=WTRREYJR014) | NO |  | 0 |  | CYT |  | YES | NO |
| 4_CDS26 | 22608 | 22733 | probable cell surface adhesin | probable cell surface adhesin [uncultured haloarchaeon] | 43% | [ABQ76136.1](http://www.ncbi.nlm.nih.gov/protein/148508354?report=genbank&log$=prottop&blast_rank=1&RID=WTRSF7U2015) | NO |  | 0 |  | CYT |  | YES | YES |
| 4_CDS29 | 22882 | 27207 | major variable surface protein | probable cell surface adhesin [uncultured haloarchaeon] | 43% | [ABQ76136.1](http://www.ncbi.nlm.nih.gov/protein/148508354?report=genbank&log$=prottop&blast_rank=1&RID=WTRSF7U2015) | NO |  | 0 |  | CYT |  | YES | NO |
| 4_CDS32 | 27872 | 29263 | putative ABC-type transporter systems, calcium-binding protein | ABC-type cobalamin/Iron(III)-siderophore transport systems, substrate-binding protein [uncultured haloarchaeon] | 94% | [ABQ76135.1](http://www.ncbi.nlm.nih.gov/protein/148508353?report=genbank&log$=prottop&blast_rank=1&RID=WTRTCV4V01R) | NO |  | 1 | S | SpI | cleavage=30-31 | YES | NO |
| 4_CDS34 | 29535 | 29648 |  | N/A |  |  | NO |  | 0 |  | CYT |  | YES | NO |
| 4_CDS35 | 29906 | 33721 | cell surface glycoprotein precursor | cell surface glycoprotein precursor [Haloquadratum walsbyi] | 29% | [WP_011570500.1](http://www.ncbi.nlm.nih.gov/protein/499889766?report=genbank&log$=prottop&blast_rank=3&RID=WTW9JH4E01R) | NO |  | 1 |  | SpI | cleavage=21-22 | YES | NO |
| 4_CDS38 | 34045 | 36930 | S-layer protein | HQ1207A cell surface glycoprotein precursor [ Haloquadratum walsbyi DSM 16790 ] | 81% | WP_011570500.1 | NO |  | 1 |  | CYT |  | YES | YES |
| 4_CDS39 | 37134 | 37304 | hypothetical protein | hypothetical protein [Haloquadratum walsbyi] | 78% | [WP_011570503.1](http://www.ncbi.nlm.nih.gov/protein/499889769?report=genbank&log$=prottop&blast_rank=1&RID=WTWAAX3V01R) | YES |  | 1 | S | SpI | cleavage=25-26 | NO | YES |
| 4_CDS40 | 37408 | 37806 | hypothetical protein | hypothetical protein [Haloquadratum walsbyi] | 96% | [WP_011570504.1](http://www.ncbi.nlm.nih.gov/protein/499889770?report=genbank&log$=prottop&blast_rank=1&RID=WTWAN1N9015) | NO |  | 4 | S | TMH |  | NO | NO |
| 4_CDS41 | 38369 | 39136 | transcription regulator-like protein | TetR family transcription regulator [Haloquadratum walsbyi DSM 16790] | 99% | [CAJ51342.1](http://www.ncbi.nlm.nih.gov/protein/403212800?report=genbank&log$=prottop&blast_rank=1&RID=WTWAY4PA014) | NO |  | 0 |  | CYT |  | NO | YES |
| 4_CDS43 | 39272 | 40900 | S-layer domain protein | S-layer domain protein [Haloquadratum walsbyi] | 90% | [WP_021055880.1](http://www.ncbi.nlm.nih.gov/protein/544617530?report=genbank&log$=prottop&blast_rank=4&RID=WTWSADBX014) | YES |  | 2 | S | SpI | cleavage=25-26 | YES | YES |
| 4_CDS44 | 40905 | 41960 | RND superfamily multidrug efflux system protein | putative exporters of the RND superfamily [Haloquadratum walsbyi] | 97% | [WP_021049932.1](http://www.ncbi.nlm.nih.gov/protein/544611581?report=genbank&log$=prottop&blast_rank=1&RID=WTWSN0CP01R) | NO |  | 6 | S | SpI | cleavage=37-38 | YES | NO |
| 5_CDS1 | 1 | 1961 | subtilisin like serine protease | subtilisin like serine protease [Haloquadratum walsbyi] | 95% | [ABQ76140.1](http://www.ncbi.nlm.nih.gov/protein/148508358?report=genbank&log$=prottop&blast_rank=1&RID=WTWSZHNG01R) | NO |  | 1 | S | SpI | cleavage=28-29 | YES | YES |
| 5_CDS2 | 2629 | 3318 | hypothetical protein | hypothetical protein [uncultured haloarchaeon eHwalsbyi559] | 98% | [ABQ76139.1](http://www.ncbi.nlm.nih.gov/protein/148508357?report=genbank&log$=prottop&blast_rank=1&RID=WTWT99DT01R) | NO |  | 0 |  | CYT |  | YES | NO |
| 5_CDS3 | 3491 | 4042 | hypothetical protein | hypothetical protein [Haloquadratum walsbyi] | 97% | [WP_011570496.1](http://www.ncbi.nlm.nih.gov/protein/499889762?report=genbank&log$=prottop&blast_rank=1&RID=WTWTPR4R014) | NO |  | 0 |  | SpII | cleavage=29-30 | YES | YES |
| 5_CDS4 | 4476 | 4694 | hypothetical protein | hypothetical protein [Haloquadratum walsbyi] | 94% | [WP_011570497.1](http://www.ncbi.nlm.nih.gov/protein/499889763?report=genbank&log$=prottop&blast_rank=1&RID=WTXZMG1H01R) | NO |  | 0 |  | CYT |  | YES | YES |
| 5_CDS5 | 4885 | 9657 | major variable surface protein | hypothetical protein [Escherichia coli] | 23% | [WP_021549794.1](http://www.ncbi.nlm.nih.gov/protein/545253082?report=genbank&log$=prottop&blast_rank=4&RID=WTY00CM1014) | YES |  | 1 | S | SpI | cleavage=32-33 | YES | YES |
| 5_CDS6 | 11750 | 12298 | probable cell surface adhesin | hypothetical protein [Haloquadratum walsbyi] | 40% | [WP_021055900.1](http://www.ncbi.nlm.nih.gov/protein/544617550?report=genbank&log$=prottop&blast_rank=1&RID=WTY0CVYX014) | NO |  | 0 |  | CYT |  | YES | NO |
| 5_CDS7 | 13134 | 13616 | IS1341 type transposase | transposase [Halobacterium salinarum] | 37% | [WP_012289618.1](http://www.ncbi.nlm.nih.gov/protein/501246600?report=genbank&log$=prottop&blast_rank=2&RID=WTY0MRXA015) | NO |  | 0 |  | CYT |  | YES | NO |
| 5_CDS8 | 14795 | 18181 | probable cell surface adhesin | hypothetical protein [Haloquadratum walsbyi] | 45% | [WP_021049551.1](http://www.ncbi.nlm.nih.gov/protein/544611200?report=genbank&log$=prottop&blast_rank=1&RID=WTY154MZ015) | YES | YES | 1 | S | SpI | cleavage=38-39 | YES | NO |
| 5_CDS9 | 18949 | 20346 | ABC type cobalamin/Iron III siderophore transport systems substrate binding protein | ABC type cobalamin/Iron III siderophore transport systems substrate binding protein [Haloquadratum walsbyi] | 94% | [ABQ76135.1](http://www.ncbi.nlm.nih.gov/protein/148508353?report=genbank&log$=prottop&blast_rank=1&RID=WTYA65C0014) | YES |  | 1 | S | SpI | cleavage=30-31 | YES | YES |
| 5_CDS10 | 20790 | 21644 | halocyanin-like protein | Beta-Ig-H3/fasciclin [uncultured haloarchaeon] | 96% | [ABQ76134.1](http://www.ncbi.nlm.nih.gov/protein/148508352?report=genbank&log$=prottop&blast_rank=1&RID=WTYBX056015) | NO |  | 0 |  | CYT |  | YES | YES |
| 5_CDS11 | 21878 | 23578 | 5' nucleotidase | 2'3'-cyclic-nucleotide 2'-phosphodiesterase [uncultured haloarchaeon] | 98% | [ABQ76132.1](http://www.ncbi.nlm.nih.gov/protein/148508350?report=genbank&log$=prottop&blast_rank=1&RID=WTYHWJNE014) | NO |  | 0 |  | SpI | cleavage=33-34 | YES | YES |
| 5_CDS12 | 23873 | 27760 | cell surface glycoprotein precursor | cell surface glycoprotein precursor [Haloquadratum walsbyi] | 29% | [WP_011570500.1](http://www.ncbi.nlm.nih.gov/protein/499889766?report=genbank&log$=prottop&blast_rank=3&RID=WTYDAEFH015) | NO |  | 1 |  | SpI | cleavage=19-20 | YES | YES |
| 5_CDS14 | 28069 | 30957 | S layer protein | cell surface glycoprotein [Haloquadratum walsbyi] | 51% | [WP_014555115.1](http://www.ncbi.nlm.nih.gov/protein/504368013?report=genbank&log$=prottop&blast_rank=8&RID=WTYNX070014) | NO |  | 1 |  | SpI | cleavage=19-20 | YES | YES |
| 5_CDS16 | 32952 | 33617 | transcriptional regulator TetR family | TetR family transcription regulator [Haloquadratum walsbyi DSM 16790] | 100% | [CAJ51342.1](http://www.ncbi.nlm.nih.gov/protein/403212800?report=genbank&log$=prottop&blast_rank=1&RID=WU2UE9J401R) | NO |  | 0 |  | CYT |  | YES | NO |
| 5_CDS17 | 33753 | 35381 | hypothetical protein | hypothetical protein [Haloquadratum walsbyi] | 99% | [WP_011570505.1](http://www.ncbi.nlm.nih.gov/protein/499889771?report=genbank&log$=prottop&blast_rank=1&RID=WU2US4SD014) | YES |  | 2 | S | SpI | cleavage=25-26 | YES | NO |
| 5_CDS18 | 35446 | 37502 | RND superfamily multidrug efflux system protein | RND transporter [Haloquadratum walsbyi] | 99% | [WP_011570506.1](http://www.ncbi.nlm.nih.gov/protein/499889772?report=genbank&log$=prottop&blast_rank=1&RID=WU2V2M0W015) | NO |  | 9 | S | SpI | cleavage=22-23 | YES | YES |
| 6_CDS1 | 2 | 460 | cell surface glycoprotein | glycoprotein gp2 [Haloquadratum walsbyi] | 99% | [WP_011570489.1](http://www.ncbi.nlm.nih.gov/protein/499889755?report=genbank&log$=prottop&blast_rank=2&RID=WU2VC4K7014) | NO |  | 1 | S | SpI | cleavage=34-35 | YES | YES |
| 6_CDS2 | 536 | 1180 | cell surface glycoprotein | cell surface glycoprotein [Haloquadratum walsbyi] | 99% | [WP_011570490.1](http://www.ncbi.nlm.nih.gov/protein/499889756?report=genbank&log$=prottop&blast_rank=1&RID=WU2VPYPY01R) | YES |  | 0 | S | SpII | cleavage=20-21 | YES | YES |
| 6_CDS04 | 1777 | 2097 | putative transcription regulator | ArsR family transcriptional regulator [Geobacillus thermoglucosidasius] | 22% | [WP_003250446.1](http://www.ncbi.nlm.nih.gov/protein/489343288?report=genbank&log$=prottop&blast_rank=6&RID=WWMTY8TP01R) | NO |  | 0 |  | CYT |  | YES | YES |
| 6_CDS05 | 2081 | 2296 | hypothetical protein | hypothetical protein [uncultured haloarchaeon] | 71% | [WP_014556634.1](http://www.ncbi.nlm.nih.gov/protein/504369532?report=genbank&log$=prottop&blast_rank=1&RID=WWENYZHC014) | NO |  | 0 |  | CYT |  | YES | YES |
| 6_CDS06 | 2299 | 2499 | hypothetical protein | N\A |  |  | NO |  | 0 |  | CYT |  | YES | YES |
| 6_CDS07 | 3332 | 6097 | subtilisin-like serine protease | subtilisin-like serine protease [Haloquadratum walsbyi] | 65% | [WP_011570501.1](http://www.ncbi.nlm.nih.gov/protein/499889767?report=genbank&log$=prottop&blast_rank=1&RID=WWF1R7WX014) | NO |  | 1 | S | SpI | cleavage=23-24 | YES | YES |
| 6_CDS08 | 6534 | 6650 | hypothetical protein | N/A |  |  | NO |  | 1 | S | CYT |  | NO | NO |
| 6_CDS11 | 6714 | 6839 | hypothetical protein | N/A |  |  | NO |  | 0 |  | CYT |  | NO | NO |
| 6_CDS12 | 6842 | 7402 | hypothetical protein | hypothetical protein [uncultured haloarchaeon] | 97% | [ABQ76139.1](http://www.ncbi.nlm.nih.gov/protein/148508357?report=genbank&log$=prottop&blast_rank=1&RID=WWF2RG8C014) | NO |  | 0 | S | CYT |  | YES | NO |
| 6_CDS14 | 7802 | 8008 | hypothetical protein | N/A |  |  | NO |  | 0 |  | CYT |  | NO | YES |
| 6_CDS15 | 8012 | 8137 | hypothetical protein | N/A |  |  | NO |  | 0 |  | CYT |  | NO | NO |
| 6_CDS17 | 8286 | 11984 | major variable surface protein | invasin [Escherichia coli] | 23% | [WP_000907433.1](http://www.ncbi.nlm.nih.gov/protein/446830177?report=genbank&log$=prottop&blast_rank=7&RID=WWFUM1UJ01R) | NO |  | 0 |  | CYT |  | NO | NO |
| 6_CDS18 | 12085 | 13089 | note=contains EF hand | hypothetical protein [Haloquadratum walsbyi] | 40% | [WP_021049551.1](http://www.ncbi.nlm.nih.gov/protein/544611200?report=genbank&log$=prottop&blast_rank=1&RID=WWFZDM4R01R) | NO |  | 0 |  | CYT |  | YES | NO |
| 6_CDS20 | 13377 | 14774 | ABC-type cobalamin/Iron(III)-siderophore transport systems, substrate-binding protein | ABC-type cobalamin/Iron(III)-siderophore transport systems, substrate-binding protein [uncultured haloarchaeon] | 89% | [ABQ76135.1](http://www.ncbi.nlm.nih.gov/protein/148508353?report=genbank&log$=prottop&blast_rank=1&RID=WWFZW750015) | YES |  | 1 | S | SpI | cleavage=30-31 | YES | NO |
| 6_CDS22 | 15303 | 18584 | cell surface glycoprotein precursor | cell surface glycoprotein precursor [Haloquadratum walsbyi] | 34% | [WP_011570499.1](http://www.ncbi.nlm.nih.gov/protein/499889765?report=genbank&log$=prottop&blast_rank=1&RID=WWG09PYV01R) | YES |  | 1 | S | SpI | cleavage=21-22 | YES | NO |
| 6_CDS23 | 19057 | 21954 | S-layer protein | RecName: Full=Cell surface glycoprotein; AltName: Full=S-layer glycoprotein; Flags: Precursor [Haloarcula japonica] | 54% | [Q9C4B4.1](http://www.ncbi.nlm.nih.gov/protein/39930912?report=genbank&log$=prottop&blast_rank=2&RID=WWG1K8CB014) | NO |  | 1 |  | CYT |  | YES | YES |
| 6_CDS24 | 22158 | 22328 | cell surface protein | N/A |  |  | YES |  | 1 | S | SpI | cleavage=25-26 | NO | YES |
| 6_CDS25 | 22431 | 22829 | hypothetical protein | hypothetical protein [Haloquadratum walsbyi] | 96% | [WP_011570504.1](http://www.ncbi.nlm.nih.gov/protein/499889770?report=genbank&log$=prottop&blast_rank=1&RID=WWG2RDZF01R) | NO |  | 4 | S | TMH |  | NO | YES |
| 6_CDS27 | 23393 | 24160 | transcription regulator-like protein | TetR family transcription regulator [Haloquadratum walsbyi DSM 16790] | 99% | [CAJ51342.1](http://www.ncbi.nlm.nih.gov/protein/403212800?report=genbank&log$=prottop&blast_rank=1&RID=WWMAWNAW014) | NO |  | 0 |  | CYT |  | YES | YES |
| 6_CDS29 | 24296 | 25924 | S-layer domain protein | hypothetical protein [Haloquadratum walsbyi] | 99% | [WP_011570505.1](http://www.ncbi.nlm.nih.gov/protein/499889771?report=genbank&log$=prottop&blast_rank=1&RID=WWMBK5DJ015) | YES |  | 2 | S | SpI | cleavage=25-26 | YES | YES |
| 6_CDS32 | 25929 | 28268 | RND superfamily multidrug efflux system protein | RND transporter [Haloquadratum walsbyi] | 99% | [WP_011570506.1](http://www.ncbi.nlm.nih.gov/protein/499889772?report=genbank&log$=prottop&blast_rank=1&RID=WWHSGRMJ015) | NO |  | 13 | S | SpI | cleavage=37-38 | YES | YES |
| 6_CDS33 | 28261 | 28758 | stress response protein | universal stress protein UspA [Haloquadratum walsbyi] | 99% | [WP_011570507.1](http://www.ncbi.nlm.nih.gov/protein/499889773?report=genbank&log$=prottop&blast_rank=1&RID=WWHTYTMG01R) | NO |  | 0 |  | CYT |  | NO | YES |
| 6_CDS34 | 28918 | 30267 | hypothetical protein | nucleotide pyrophosphatase [Haloquadratum walsbyi] | 99% | [WP_014555121.1](http://www.ncbi.nlm.nih.gov/protein/504368019?report=genbank&log$=prottop&blast_rank=1&RID=WWHUBYU901R) | NO |  | 0 |  | CYT |  | YES | NO |
| 6_CDS35 | 30532 | 31050 | hypothetical protein | hypothetical protein [Haloquadratum walsbyi] | 99% | [WP_011570509.1](http://www.ncbi.nlm.nih.gov/protein/499889775?report=genbank&log$=prottop&blast_rank=1&RID=WWHX7T01014) | NO |  | 2 | S | TMH |  | YES | YES |
| 6_CDS36 | 31203 | 31676 | hypothetical protein | hypothetical protein [Haloquadratum walsbyi] | 99% | [WP_011570510.1](http://www.ncbi.nlm.nih.gov/protein/499889776?report=genbank&log$=prottop&blast_rank=1&RID=WWHXBJ7E015) | NO |  | 0 |  | CYT |  | YES | YES |
| 6_CDS38 | 31676 | 33463 | family X DNA-dependent DNA polymerase | DNA polymerase X family [Bacillus subtilis E1] | 33% | [CCU59360.1](http://www.ncbi.nlm.nih.gov/protein/659925025?report=genbank&log$=prottop&blast_rank=12&RID=WWHXYU1T015) | NO |  | 0 |  | CYT |  | YES | yES |
| 6_CDS40 | 33583 | 34134 | hypothetical protein | hypothetical protein [Haloquadratum walsbyi] | 99% | [WP_011570512.1](http://www.ncbi.nlm.nih.gov/protein/499889778?report=genbank&log$=prottop&blast_rank=1&RID=WWKUV402015) | NO |  | 0 |  | CYT |  | YES | NO |
| 6_CDS42 | 34161 | 35036 | hypothetical protein | hypothetical protein [Haloquadratum walsbyi] | 99% | [WP_011570513.1](http://www.ncbi.nlm.nih.gov/protein/499889779?report=genbank&log$=prottop&blast_rank=1&RID=WWKV7MU3015) | NO |  | 3 | S | CYT |  | YES | NO |
| 6_CDS43 | 35161 | 35280 | hypothetical protein | N/A |  |  | NO |  | 0 |  | CYT |  | NO | NO |
| 6_CDS44 | 35329 | 35700 | hypothetical protein | hypothetical protein [Haloquadratum walsbyi] | 99% | [WP_011570514.1](http://www.ncbi.nlm.nih.gov/protein/499889780?report=genbank&log$=prottop&blast_rank=1&RID=WWKW180X014) | NO |  | 0 |  | CYT |  | YES | NO |
| 6_CDS47 | 35985 | 36590 | protease | conserved hypothetical protein [Haloquadratum walsbyi DSM 16790] | 100% | [CAJ51353.2](http://www.ncbi.nlm.nih.gov/protein/469662328?report=genbank&log$=prottop&blast_rank=1&RID=WWKWCEA7014) | NO |  | 3 | S | CYT |  | NO | NO |
| 7_CDS1 | 1 | 849 | type II secretion system transmembrane protein | hypothetical protein [Haloquadratum walsbyi] | 100% | [WP_011570483.1](http://www.ncbi.nlm.nih.gov/protein/499889749?report=genbank&log$=prottop&blast_rank=1&RID=WWKWSDP0014) | NO |  | 4 |  | CYT |  | YES | NO |
| 7_CDS2 | 849 | 2813 | type II secretion system transmembrane protein/IV secretion system proteins VirB11/TadA ATPase | type II/IV secretion system ATPase [Haloquadratum walsbyi] | 99% | [WP_011570484.1](http://www.ncbi.nlm.nih.gov/protein/499889750?report=genbank&log$=prottop&blast_rank=1&RID=WWKX3KBE015) | NO |  | 0 |  | CYT |  | YES | NO |
| 7_CDS3 | 3018 | 3449 | hypothetical protein | hypothetical protein [Haloquadratum walsbyi] | 97% | [WP_014555112.1](http://www.ncbi.nlm.nih.gov/protein/504368010?report=genbank&log$=prottop&blast_rank=1&RID=WWP1UPDN014) | NO |  | 0 |  | CYT |  | YES | NO |
| 7_CDS4 | 3488 | 4345 | dentin sialophosphoprotein precursor | HQ1192A dentin sialophosphoprotein precursor [ Haloquadratum walsbyi DSM 16790 ] | 98% | [WP_011570486.1](http://www.ncbi.nlm.nih.gov/protein/499889752?report=genbank&log$=prottop&blast_rank=1&RID=WWP2FXRW01R) | NO |  | 0 |  | CYT |  | YES | NO |
| 7_CDS5 | 4759 | 8619 | cell surface glycoprotein | probable cell surface glycoprotein [uncultured haloarchaeon] | 92% | [ABQ76148.1](http://www.ncbi.nlm.nih.gov/protein/148508366?report=genbank&log$=prottop&blast_rank=1&RID=WWP32BKA015) | NO |  | 2 | S | CYT |  | YES | NO |
| 7_CDS6 | 9362 | 10189 | ISH9 type transposase | transposase [Haloquadratum walsbyi] | 99% | [WP_011571298.1](http://www.ncbi.nlm.nih.gov/protein/499890564?report=genbank&log$=prottop&blast_rank=1&RID=WWP3CVS5014) | NO |  | 0 |  | CYT |  | YES | NO |
| 7_CDS7 | 10390 | 12223 | hypothetical protein | hypothetical protein [uncultured haloarchaeon] | 96% | [ABQ76147.1](http://www.ncbi.nlm.nih.gov/protein/148508365?report=genbank&log$=prottop&blast_rank=1&RID=WWP43XPX01R) | NO |  | 1 | S | SpI | cleavage=34-35 | YES | NO |
| 7_CDS8 | 12436 | 13191 | hypothetical protein | N/A |  |  | NO |  | 0 |  | CYT |  | YES | NO |
| 7_CDS9 | 13613 | 19118 | halomucin2 | hmu2 cell surface glycoprotein [ Haloquadratum walsbyi DSM 16790 ] | 93% | [WP_011570491.1](http://www.ncbi.nlm.nih.gov/protein/499889757?report=genbank&log$=prottop&blast_rank=1&RID=WWR34ZGE01R) | NO |  | 1 |  | CYT |  | YES | YES |
| 7_CDS12 | 19237 | 19887 | cell surface glycoprotein | cell surface glycoprotein [Haloquadratum walsbyi] | 59% | [WP_011570490.1](http://www.ncbi.nlm.nih.gov/protein/499889756?report=genbank&log$=prottop&blast_rank=8&RID=WWR3G4SG01R) | YES |  | 0 | S | SpII | cleavage=20-21 | YES | NO |
| 7_CDS13 | 20069 | 20686 | hypothetical protein | hypothetical protein OSG_eHP16_00150 [Environmental Halophage eHP-16] | 49% | [AFH22122.1](http://www.ncbi.nlm.nih.gov/protein/383397333?report=genbank&log$=prottop&blast_rank=4&RID=WWR3T8CH01R) | NO |  | 0 |  | CYT |  | YES | YES |
| 7_CDS14 | 20788 | 21063 | hypothetical protein | hypothetical protein [uncultured virus] | 96% | [ADE29248.1](http://www.ncbi.nlm.nih.gov/protein/292496147?report=genbank&log$=prottop&blast_rank=1&RID=WWR44SK8014) | NO |  | 0 |  | CYT |  | NO | NO |
| 7_CDS15 | 21805 | 24600 | putative long tail fiber proximal subunit | hypothetical protein [candidate division NKB19 bacterium JGI 0000077-D07] | 33% | [WP_029951360.1](http://www.ncbi.nlm.nih.gov/protein/661256034?report=genbank&log$=prottop&blast_rank=2&RID=WWRAZNC9014) | YES |  | 1 | S | SpI | cleavage=26-27 | YES | YES |
| 7_CDS16 | 24960 | 26354 | ABC type cobalamin | ABC-type cobalamin/Iron(III)-siderophore transport systems, substrate-binding protein [uncultured haloarchaeon] | 90% | [ABQ76135.1](http://www.ncbi.nlm.nih.gov/protein/148508353?report=genbank&log$=prottop&blast_rank=1&RID=WWRBGEZN014) | YES |  | 1 | S | SpI | cleavage=30-31 | YES | YES |
| 7_CDS18 | 27120 | 28088 | hypothetical protein | N/A |  |  | NO |  | 0 |  | CYT |  | YES | NO |
| 7_CDS19 | 28081 | 29778 | PKD domain containing protein | PKD domain-containing protein [Candidatus Magnetoglobus multicellularis str. Araruama] | 25% | [ETR69119.1](http://www.ncbi.nlm.nih.gov/protein/571788127?report=genbank&log$=prottop&blast_rank=3&RID=WWRC4GXC01R) | NO | YES | 0 |  | SpI | cleavage=21-22 | YES | NO |
| 7_CDS20 | 29784 | 31331 | PKD domain containing protein | PKD domain-containing protein [Halorhabdus utahensis] | 40% | [WP_015788362.1](http://www.ncbi.nlm.nih.gov/protein/506268587?report=genbank&log$=prottop&blast_rank=1&RID=WWY3MANP015) | NO |  | 1 |  | CYT |  | NO | YES |
| 7_CDS21 | 31818 | 32117 | hypothetical protein | hypothetical protein [Haloquadratum sp. J07HQX50] | 54% | [WP_021059155.1](http://www.ncbi.nlm.nih.gov/protein/544620819?report=genbank&log$=prottop&blast_rank=6&RID=WWY42T3Y015) | NO |  | 0 |  | CYT |  | YES | YES |
| 7_CDS22 | 32265 | 32603 | hypothetical protein | N/A |  |  | NO |  | 0 |  | CYT |  | YES | NO |
| 7_CDS23 | 32761 | 33225 | putative phage primase | phage/plasmid primase, P4 family protein [Haloferax sulfurifontis] | 33% | [WP_007273992.1](http://www.ncbi.nlm.nih.gov/protein/494484519?report=genbank&log$=prottop&blast_rank=5&RID=WWY4X0Z001R) | NO |  | 0 |  | CYT |  | YES | YES |
| 7_CDS24 | 34007 | 34417 | hypothetical protein | hypothetical protein [Natronococcus jeotgali] | 40% | [WP_008424846.1](http://www.ncbi.nlm.nih.gov/protein/495700267?report=genbank&log$=prottop&blast_rank=2&RID=WWY5PJNA014) | NO |  | 0 |  | CYT |  | YES | YES |
| 7_CDS25 | 35440 | 35977 | FG-GAP_2 repeat containing protein | PKD domain-containing protein [Halorhabdus utahensis] | 48% | [WP_015788362.1](http://www.ncbi.nlm.nih.gov/protein/506268587?report=genbank&log$=prottop&blast_rank=3&RID=WWYZJPKK014) | NO |  | 0 |  | CYT |  | NO | NO |
| 9_CDS1 | 4 | 1623 | hypothetical protein | hypothetical protein [uncultured haloarchaeon] | 80% | [ABQ76147.1](http://www.ncbi.nlm.nih.gov/protein/148508365?report=genbank&log$=prottop&blast_rank=1&RID=WYW43PBV014) | YES |  | 1 | S | SpI | cleavage=34-35 | YES | NO |
| 9_CDS3 | 1819 | 2463 | cell surface glycoprotein | cell surface glycoprotein [Haloquadratum walsbyi] | 96% | [WP_011570490.1](http://www.ncbi.nlm.nih.gov/protein/499889756?report=genbank&log$=prottop&blast_rank=1&RID=WYW4YFBK014) | YES |  | 0 | S | SpII | cleavage=20-21 | YES | NO |
| 9_CDS04 | 2679 | 2840 | hypothetical protein | N/A |  |  | NO |  | 1 | S | CYT |  | YES | NO |
| 9_CDS5 | 2806 | 3090 | ribbon helix helix protein | conserved hypothetical protein [Albugo laibachii Nc14] | 21% | [CCA15078.1](http://www.ncbi.nlm.nih.gov/protein/325180673?report=genbank&log$=prottop&blast_rank=1&RID=WYW5YR2E014) | NO |  | 0 |  | CYT |  | NO | NO |
| 9_CDS6 | 3199 | 3324 | hypothetical protein | N/A |  |  | NO |  | 0 |  | CYT |  | NO | YES |
| 9_CDS10 | 3816 | 6683 | GLUG domain protein | GLUG domain protein [Natronorubrum bangense] | 31% | [WP_006067186.1](http://www.ncbi.nlm.nih.gov/protein/492959101?report=genbank&log$=prottop&blast_rank=7&RID=WYW6RYHS014) | NO |  | 0 |  | CYT |  | YES | YES |
| 9_CDS11 | 6649 | 6846 | hypothetical protein | N/A |  |  | NO |  | 0 |  | CYT |  | YES | NO |
| 9_CDS14 | 7236 | 7430 | hypothetical protein | N/A |  |  | NO |  | 0 |  | CYT |  | YES | NO |
| 9_CDS15 | 7675 | 8259 | hypothetical protein | hypothetical protein HQ_1199A [Haloquadratum walsbyi DSM 16790] | 95% | [CAJ51328.2](http://www.ncbi.nlm.nih.gov/protein/403212799?report=genbank&log$=prottop&blast_rank=1&RID=WYWEYYTP01R) | NO |  | 0 |  | SpII | cleavage=28-29 | YES | NO |
| 9_CDS16 | 8485 | 8631 | hypothetical protein | subtilisin-like serine protease [uncultured haloarchaeon] | 67% | [ABQ76140.1](http://www.ncbi.nlm.nih.gov/protein/148508358?report=genbank&log$=prottop&blast_rank=1&RID=WYWF833E01R) | NO |  | 0 |  | CYT |  | YES | YES |
| 9_CDS18 | 8810 | 8932 | hypothetical protein | N/A |  |  | NO |  | 0 |  | CYT |  | YES | NO |
| 9_CDS20 | 9726 | 10046 | putative transcription regulator | hypothetical protein [Haloquadratum walsbyi] | 84% | [WP_021053633.1](http://www.ncbi.nlm.nih.gov/protein/544615283?report=genbank&log$=prottop&blast_rank=1&RID=WYWFX5KY01R) | NO |  | 0 |  | CYT |  | YES | NO |
| 9_CDS21 | 10030 | 10227 | hypothetical protein | hypothetical protein [uncultured haloarchaeon] | 84% | [ABQ76143.1](http://www.ncbi.nlm.nih.gov/protein/148508361?report=genbank&log$=prottop&blast_rank=1&RID=WYZB5JMR01R) | NO |  | 0 |  | CYT |  | YES | NO |
| 9_CDS22 | 10284 | 11036 | hypothetical protein | hypothetical protein [Haloquadratum walsbyi] | 66% | [WP_011572909.1](http://www.ncbi.nlm.nih.gov/protein/499892175?report=genbank&log$=prottop&blast_rank=1&RID=WYZBEB3U01R) | NO |  | 5 |  | CYT |  | NO | NO |
| 9_CDS24 | 12324 | 12497 | hypothetical protein | hypothetical protein [Natrinema pellirubrum] | 30% | [WP_006179706.1](http://www.ncbi.nlm.nih.gov/protein/493181419?report=genbank&log$=prottop&blast_rank=4&RID=WYZC1V4301R) | YES |  | 1 | S | SpI | cleavage=28-29 | NO | NO |
| 9_CDS25 | 12639 | 13301 | hypothetical protein | N/A |  |  | NO |  | 0 |  | SpI | cleavage=17-18 | YES | NO |
| 9_CDS26 | 14214 | 16400 | subtilisin-like serine protease | ike serine protease [uncultured haloarchaeon] | 86% | [ABQ76140.1](http://www.ncbi.nlm.nih.gov/protein/148508358?report=genbank&log$=prottop&blast_rank=1&RID=WYZCH2SW015) | NO |  | 1 | S | SpI | cleavage=28-29 | YES | NO |
| 9_CDS28 | 17297 | 20386 | major variable surface protein | probable cell surface adhesin [uncultured haloarchaeon] | 27% | [ABQ76136.1](http://www.ncbi.nlm.nih.gov/protein/148508354?report=genbank&log$=prottop&blast_rank=3&RID=WYZCU141015) | NO |  | 1 | S | SpI | cleavage=34-35 | YES | NO |
| 9_CDS29 | 21417 | 21635 | transposase, IS605 OrfB family | transposase, IS605 OrfB family, central region [uncultured archaeon A07HR60] | 83% | [WP_023504369.1](http://www.ncbi.nlm.nih.gov/protein/558591648?report=genbank&log$=prottop&blast_rank=1&RID=WYZH9WYY014) | NO |  | 0 |  | CYT |  | NO | YES |
| 9_CDS31 | 21708 | 21827 | hypothetical protein | N/A |  |  | NO |  | 0 |  | CYT |  | YES | NO |
| 9_CDS33 | 22121 | 22264 | transposase, IS605 OrfB family protein | transposase, IS605 OrfB family protein [Halosarcina pallida] | 65% | [WP_008388210.1](http://www.ncbi.nlm.nih.gov/protein/495663631?report=genbank&log$=prottop&blast_rank=1&RID=WYZJ09EM015) | NO |  | 0 |  | CYT |  | NO | YES |
| 9_CDS34 | 22496 | 22726 | putative transcription regulator | ranscriptional regulator of a riboflavin/FAD biosynthetic operon [Natrinema pellirubrum] | 40% | [WP_007743318.1](http://www.ncbi.nlm.nih.gov/protein/495017310?report=genbank&log$=prottop&blast_rank=1&RID=WYZJB8UC014) | NO |  | 0 |  | CYT |  | NO | YES |
| 9_CDS36 | 23689 | 24807 | transposase | transposase [Halorhabdus tiamatea] | 89% | [WP_008528511.1](http://www.ncbi.nlm.nih.gov/protein/495803932?report=genbank&log$=prottop&blast_rank=1&RID=WYZYZY7W01R) | NO |  | 0 |  | CYT |  | NO | NO |
| 9_CDS37 | 25261 | 25944 | hypothetical protein | N/A |  |  | NO |  | 0 |  | CYT |  | YES | NO |
| 9_CDS41 | 26833 | 26961 | hypothetical protein | N/A |  |  | NO |  | 0 |  | CYT |  | NO | NO |
| 9_CDS42 | 27113 | 27262 | IS1341-type transposase | IS1341-type transposase [Haloferax elongans] | 64% | [WP_008327113.1](http://www.ncbi.nlm.nih.gov/protein/495602534?report=genbank&log$=prottop&blast_rank=1&RID=WZ06NV02015) | NO |  | 0 |  | CYT |  | NO | NO |
| 9_CDS45 | 28015 | 28614 | hypothetical protein | hypothetical protein [Haloquadratum walsbyi] | 39% | [WP_021055900.1](http://www.ncbi.nlm.nih.gov/protein/544617550?report=genbank&log$=prottop&blast_rank=1&RID=WZ070XRH014) | YES |  | 0 | S | SpI | cleavage=20-21 | YES | NO |
| 9_CDS46 | 28751 | 28900 | hypothetical protein | N/A |  |  | NO |  | 0 |  | CYT |  | YES | NO |
| 9_CDS50 | 30448 | 33342 | probable cell surface adhesin | probable cell surface adhesin [uncultured haloarchaeon] | 27% | [ABQ76136.1](http://www.ncbi.nlm.nih.gov/protein/148508354?report=genbank&log$=prottop&blast_rank=1&RID=WZ0FZ4HD01R) | YES |  | 0 | S | SpI | cleavage=18-19 | YES | NO |
| 9_CDS51 | 33927 | 34040 | hypothetical protein |  |  |  | NO |  | 0 |  | CYT |  | NO | NO |
| 9_CDS52 | 34165 | 35562 | ABC-type cobalamin Iron(III)-siderophore transport systems, substrate-binding protein | ABC-type cobalamin/Iron(III)-siderophore transport systems, substrate-binding protein [uncultured haloarchaeon] | 94% | [ABQ76135.1](http://www.ncbi.nlm.nih.gov/protein/148508353?report=genbank&log$=prottop&blast_rank=1&RID=WZ0GB0GC01R) | YES |  | 1 | S | SpI | cleavage=30-31 | YES | YES |
| 9_CDS53 | 35874 | 36860 | halocyanin-like protein | ATPases of the AAA+ class [Agarivorans albus] | 29% | [WP_016400431.1](http://www.ncbi.nlm.nih.gov/protein/511819312?report=genbank&log$=prottop&blast_rank=15&RID=WZ0GG8PR015) | NO | YES | 1 |  | SpI | cleavage=35-36 | YES | NO |
| 12_CDS01 | 211 | 1755 | probable cell surface adhesin | Ig domain-containing protein [Candidatus Solibacter usitatus] | 27% | [WP_011685033.1](http://www.ncbi.nlm.nih.gov/protein/500004315?report=genbank&log$=prottop&blast_rank=1&RID=WZ0ZT62E01R) | NO |  | 0 |  | CYT |  | YES | YES |
| 12_CDS03 | 1712 | 2782 | probable surface adhesin | cell surface adhesin [Haloquadratum walsbyi] | 68% | [WP_011570498.1](http://www.ncbi.nlm.nih.gov/protein/499889764?report=genbank&log$=prottop&blast_rank=1&RID=WZ181A8B01R) | NO |  | 0 |  | CYT |  | YES | NO |
| 12_CDS06 | 3138 | 3335 | IS1341-type transposase | IS1341-type transposase [Natronomonas moolapensis] | 71% | [WP_015409578.1](http://www.ncbi.nlm.nih.gov/protein/505222476?report=genbank&log$=prottop&blast_rank=1&RID=WZ10DAYX01R) | NO |  | 0 |  | CYT |  | NO | YES |
| 12_CDS7 | 3693 | 4118 | transposase | transposase, IS605 OrfB family protein [Natronobacterium gregoryi] | 40% | [WP_005578559.1](http://www.ncbi.nlm.nih.gov/protein/491745426?report=genbank&log$=prottop&blast_rank=1&RID=WZ146MTP01R) | NO |  | 0 |  | CYT |  | NO | NO |
| 12_CDS08 | 4469 | 4609 | IS1341-type transposase | transposase [Haloquadratum walsbyi] | 63% | [WP_011571134.1](http://www.ncbi.nlm.nih.gov/protein/499890400?report=genbank&log$=prottop&blast_rank=1&RID=WZ16FUDC01R) | NO |  | 0 |  | CYT |  | NO | YES |
| 12_CDS09 | 4740 | 4898 | IS1341-type transposase | IS1341-type transposase [Haloarcula californiae] | 51% | [WP_007188085.1](http://www.ncbi.nlm.nih.gov/protein/494342814?report=genbank&log$=prottop&blast_rank=1&RID=WZ16PH6701R) | NO |  | 0 |  | CYT |  | NO | YES |
| 12_CDS10 | 5134 | 5964 | ISH9-type transposase ISHwa3 | transposase [Haloferax volcanii] | 96% | [WP_004043159.1](http://www.ncbi.nlm.nih.gov/protein/490142817?report=genbank&log$=prottop&blast_rank=1&RID=WZ18NNDM01R) | NO |  | 0 |  | CYT |  | NO | NO |
| 12_CDS12 | 6512 | 7162 | cell surface protein | MULTISPECIES: flagellar basal body rod protein FlgG [Rhodobacter] | 25% | [WP_002720227.1](http://www.ncbi.nlm.nih.gov/protein/488807821?report=genbank&log$=prottop&blast_rank=4&RID=WZ18XDAV01R) | NO |  | 1 | S | SpI | cleavage=35-36 | YES | NO |
| 12_CDS14 | 7543 | 7818 | hypothetical protein | hypothetical protein [Haloquadratum walsbyi] | 77% | [WP_021051423.1](http://www.ncbi.nlm.nih.gov/protein/544613072?report=genbank&log$=prottop&blast_rank=1&RID=WZ1C7S3601R) | NO |  | 0 |  | CYT |  | YES | NO |
| 12_CDS17 | 7856 | 8440 | IS1341-type transposase | transposase, IS605 OrfB family protein [Haloarcula amylolytica] | 85% | [WP_008311782.1](http://www.ncbi.nlm.nih.gov/protein/495587203?report=genbank&log$=prottop&blast_rank=1&RID=WZ1CM7ZX015) | NO |  | 0 |  | CYT |  | YES | NO |
| 12_CDS18 | 8450 | 8566 | hypothetical protein | hypothetical protein [Haloquadratum walsbyi] | 63% | [WP_021052965.1](http://www.ncbi.nlm.nih.gov/protein/544614614?report=genbank&log$=prottop&blast_rank=1&RID=WZ1CXYKB015) | NO |  | 0 |  | CYT |  | YES | NO |
| 12_CDS19 | 8763 | 9242 | IS1341-type transposase | transposase [Haloquadratum walsbyi] | 74% | [WP_011571134.1](http://www.ncbi.nlm.nih.gov/protein/499890400?report=genbank&log$=prottop&blast_rank=1&RID=WZ1E4G2X015) | NO |  | 0 |  | CYT |  | YES | YES |
| 12_CDS21 | 9798 | 10241 | IS605-type transposase | transposase, IS605 OrfB family, central region [uncultured archaeon A07HR60] | 87% | [WP_023506403.1](http://www.ncbi.nlm.nih.gov/protein/558593693?report=genbank&log$=prottop&blast_rank=1&RID=WZ1EDEPN015) | NO |  | 0 |  | CYT |  | YES | NO |
| 12_CDS23 | 10553 | 11008 | IS1341-type transposase | transposase [Halobiforma lacisalsi] | 34% | [WP_007141196.1](http://www.ncbi.nlm.nih.gov/protein/494235896?report=genbank&log$=prottop&blast_rank=2&RID=WZ1J15Z5014) | NO |  | 0 |  | CYT |  | YES | NO |
| 12_CDS24 | 11135 | 11284 | hypothetical protein | hypothetical protein [Haloquadratum sp. J07HQX50] | 59% | [WP_021057028.1](http://www.ncbi.nlm.nih.gov/protein/544618691?report=genbank&log$=prottop&blast_rank=1&RID=WZ1N8WSY01R) | NO |  | 0 |  | CYT |  | NO | NO |
| 12_CDS27 | 11719 | 15030 | cell surface protein | cell surface glycoprotein [Halorhabdus tiamatea] | 26% | [WP_020936044.1](http://www.ncbi.nlm.nih.gov/protein/529147086?report=genbank&log$=prottop&blast_rank=7&RID=WZ1PZVSF014) | YES |  | 0 | S | SpI | cleavage=18-19 | YES | NO |
| 12_CDS28 | 15798 | 17192 | ABC-type cobalamin Iron(III)-siderophore transport systems, substrate-binding protein | ABC transporter substrate-binding protein [Thermosediminibacter oceani] | 24% | [WP_013276210.1](http://www.ncbi.nlm.nih.gov/protein/503041234?report=genbank&log$=prottop&blast_rank=1&RID=WZ1S72PK014) | YES |  | 1 | S | SpI | cleavage=30-31 | YES | NO |
| 12_CDS29 | 17507 | 18493 | halocyanin-like protein | ATPases of the AAA+ class [Agarivorans albus] | 29% | [WP_016400431.1](http://www.ncbi.nlm.nih.gov/protein/511819312?report=genbank&log$=prottop&blast_rank=20&RID=WZ1T731801R) | NO | YES | 1 |  | SpI | cleavage=35-36 | YES | NO |
| 12_CDS30 | 18727 | 19593 | 5' nucleotidase | 5'-nucleotidase domain-containing protein [Oceanithermus profundus] | 41% | [WP_013457513.1](http://www.ncbi.nlm.nih.gov/protein/503222852?report=genbank&log$=prottop&blast_rank=1&RID=WZ38GW30015) | NO |  | 0 |  | CYT |  | YES | NO |
| 12_CDS31 | 19590 | 20426 | 5'-nucleotidase | 5'-nucleotidase [Dictyoglomus thermophilum] | 26% | [WP_012548262.1](http://www.ncbi.nlm.nih.gov/protein/501543146?report=genbank&log$=prottop&blast_rank=1&RID=WZ38S5YY015) | NO |  | 0 |  | SpI | cleavage=33-34 | YES | YES |
| 12_CDS32 | 20712 | 24626 | cell surface glycoprotein precursor | cell surface glycoprotein precursor [Haloquadratum walsbyi] | 37% | [WP_011570499.1](http://www.ncbi.nlm.nih.gov/protein/499889765?report=genbank&log$=prottop&blast_rank=2&RID=WZ39764B01R) | NO |  | 1 |  | SpI | cleavage=22-23 | YES | YES |
| 12_CDS33 | 24950 | 25330 | hypothetical protein | hypothetical protein [Haloquadratum walsbyi] | 46% | [WP_021055884.1](http://www.ncbi.nlm.nih.gov/protein/544617534?report=genbank&log$=prottop&blast_rank=1&RID=WZ39GVCJ01R) | NO |  | 0 |  | SpI | cleavage=19-20 | YES | NO |
| 12_CDS34 | 25281 | 27821 | S-layer protein | cell surface glycoprotein [Haloarcula japonica] | 54% | [WP_004592221.1](http://www.ncbi.nlm.nih.gov/protein/490729832?report=genbank&log$=prottop&blast_rank=2&RID=WZ39YP0E01R) | NO |  | 1 |  | CYT |  | YES | NO |
| 12_CDS35 | 28025 | 28195 | hypothetical protein | N/A |  |  | YES |  | 1 | S | SpI | cleavage=25-26 | NO | NO |
| 12_CDS36 | 28298 | 28696 | hypothetical protein | hypothetical protein [Haloquadratum walsbyi] | 100% | [WP_011570504.1](http://www.ncbi.nlm.nih.gov/protein/499889770?report=genbank&log$=prottop&blast_rank=1&RID=WZ3H94UE015) | NO |  | 4 | S | TMH |  | NO | YES |
| 12_CDS37 | 29789 | 30556 | transcription regulator-like protein | transcriptional regulator [Haloquadratum walsbyi] | 67% | [WP_021055881.1](http://www.ncbi.nlm.nih.gov/protein/544617531?report=genbank&log$=prottop&blast_rank=1&RID=WZ3S83D0014) | NO |  | 0 |  | CYT |  | YES | NO |
| 12_CDS39 | 30731 | 32320 | hypothetical protein | hypothetical protein [Haloquadratum walsbyi] | 99% | [WP_011570505.1](http://www.ncbi.nlm.nih.gov/protein/499889771?report=genbank&log$=prottop&blast_rank=1&RID=WZ3SX6H3014) | NO |  | 1 |  | CYT |  | YES | YES |
| 12_CDS40 | 32385 | 33695 | RND superfamily multidrug efflux system protein | putative exporters of the RND superfamily [Haloquadratum walsbyi] | 97% | [WP_021049932.1](http://www.ncbi.nlm.nih.gov/protein/544611581?report=genbank&log$=prottop&blast_rank=1&RID=WZ40Y4M5015) | NO |  | 6 | S | SpI | cleavage=22-23 | YES | NO |
