# Supplementary material for: Diversity of the cell-wall associated genomic island of the archaeon Haloquadratum walsbyi
Source: BMC Genomics. 2015 Aug 13;16(1):603. doi: 10.1186/s12864-015-1794-8 (PMC4535781; doi:10.1186/s12864-015-1794-8)
Supplement: Additional file 5: — Average frequencies of individual amino acids in ORFs found in the eHwalsbyiGI1s studied here, eHwalsbyi559 [ 10 ] (blue), and genomes of H. walsbyi HBSQ001 and C23 (red). (PPTX 49 kb) [file 12864_2015_1794_MOESM5_ESM.pptx]

## Slide 1
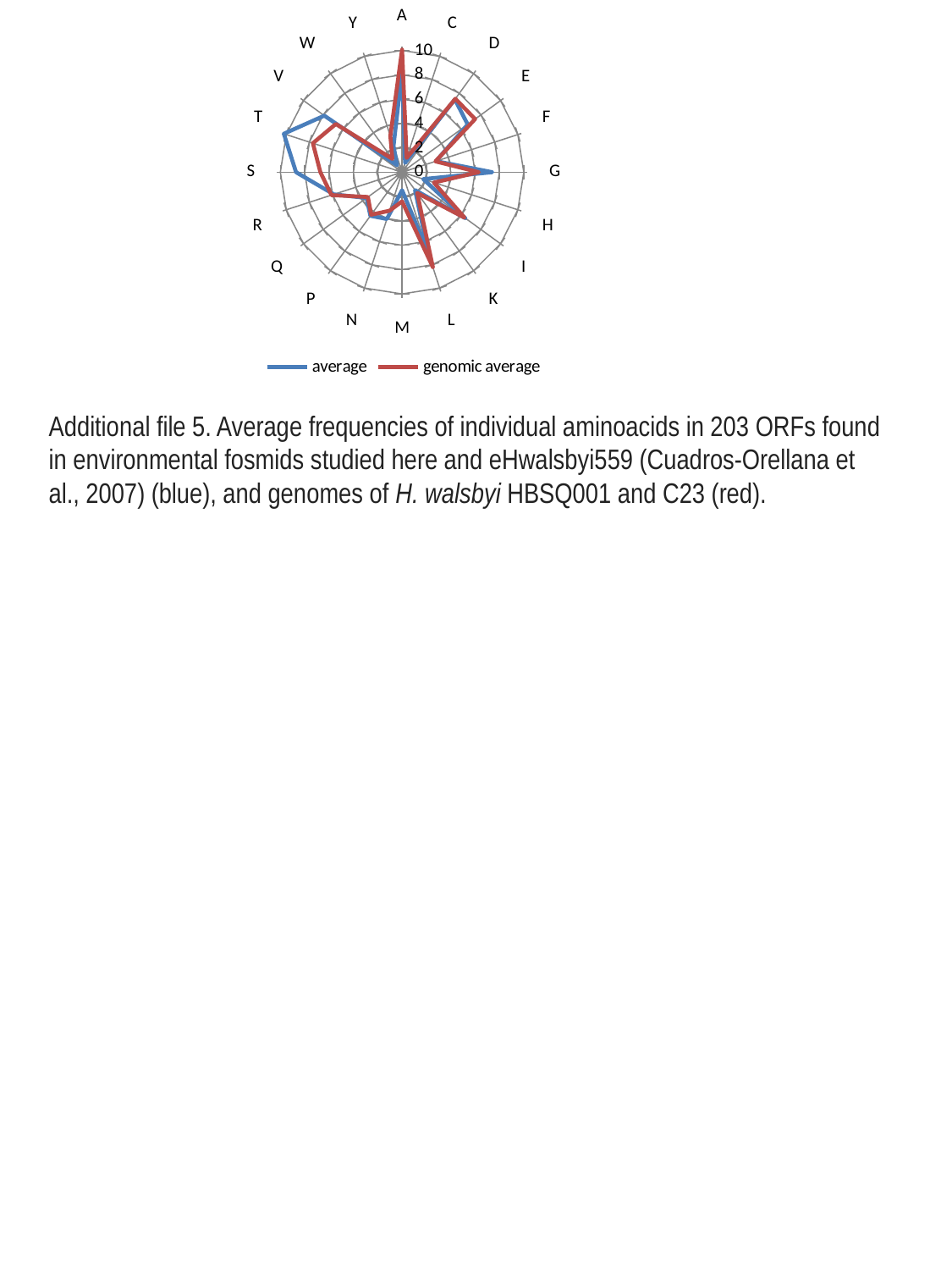

### Chart
| Category | average | genomic average |
|---|---|---|
| A | 8.342 | 10.065000000000005 |
| C | 0.807 | 1.228 |
| D | 7.387999999999997 | 7.453 |
| E | 6.664999999999996 | 7.419 |
| F | 2.9319999999999986 | 2.8859999999999997 |
| G | 7.418 | 6.302999999999997 |
| H | 1.833 | 2.751 |
| I | 6.415 | 6.3439999999999985 |
| K | 1.8169999999999993 | 2.117 |
| L | 7.147999999999997 | 8.197999999999999 |
| M | 1.478 | 2.4 |
| N | 4.042 | 3.3289999999999997 |
| P | 4.427999999999997 | 4.317999999999997 |
| Q | 3.607 | 3.468 |
| R | 5.892999999999997 | 6.096 |
| S | 8.71 | 6.711 |
| T | 10.21 | 7.702 |
| V | 7.894999999999997 | 6.727999999999997 |
| W | 0.7050000000000003 | 1.356 |
| Y | 2.2680000000000002 | 3.126 |Additional file 5. Average frequencies of individual aminoacids in 203 ORFs found in environmental fosmids studied here and eHwalsbyi559 (Cuadros-Orellana et al., 2007) (blue), and genomes of H. walsbyi HBSQ001 and C23 (red).
