# Supplementary material for: Diversity of the cell-wall associated genomic island of the archaeon Haloquadratum walsbyi
Source: BMC Genomics. 2015 Aug 13;16(1):603. doi: 10.1186/s12864-015-1794-8 (PMC4535781; doi:10.1186/s12864-015-1794-8)
Supplement: Additional file 6: — Localisation, length and consensus sequence of repeats detected in this study using XSTREAM (DOCX 31 kb) [file 12864_2015_1794_MOESM6_ESM.docx]

# Additional file 6. Localisation, length and consensus sequence of repeats detected in this study using XSTREAM

|  | Repeat charasteristic | | | | |
| --- | --- | --- | --- | --- | --- |
| Fosmid number, gene position and annotation | number. | positon | length | copy number | Consensus sequence |
| 1_CDS30  cell surface glycoprotein | 1 | 253-398 | 62 | 2.35 | TS[TP,5]SPTPSPTATPTQ[TP,3]LPTPAPTNT  [P,3]TSA[PT,2]A[TP,2]TL[TP,3] |
| 7_CDS20  PKD domain containing protein | 2 | 349-470 | 54 | 2.26 | GSAYVFSRSEGAWS[Q,3]KLTASDGAS[D,2]  RFGFPVGLS[D,2]G[T,2]ALIG[A,2]G[D,3]LT |
| 7_CDS5  cell surface glycoprotein | 3 | 288-370 | 32 | 2.59 | PTPS[PT,2][P,2]ATA[TP,4]SPTPAPTAT[P,2] |
| 1_CDS30  cell surface glycoprotein | 4 | 218-296 | 30 | 2.63 | TPTLTPTQ[TP,11] |
| 7_CDS15  putative long tail fiber  proximal subunit | 5 | 553-633 | 27 | 3.00 | ATASGNVTGESDV[G,2]LVGT[N,2][G,2]TIQN |
| 4_CDS1  cell surface glycoprotein | 6 | 77-190 | 18 | 6.33 | [TP,5]S[PT,3]P |
| eHwalsbyi559  subtilisin-like serine protease | 7 | 37-70 | 17 | 2.00 | GEIGITELGDAGRAFAE |
| 5_CDS14  S layer_protein | 8 | 890-923 | 15 | 2.27 | T[E,2]TATPEPTAT[E,2]P |
| 6_CDS23  S-layer_protein |  | 893-931 | 15 | 2.60 |  |
| 12_CDS34  S-layer_protein |  | 774-807 | 15 | 2.27 |  |
| 4_CDS38  S-layer_protein | 9 | 895-931 | 14 | 2.64 | TPEPA[T,2][E,2][PT,2]A |
| 5_CDS12  cell surface  glycoprotein precursor | 10 | 67-95 | 14 | 2.07 | [TE,2][TP,3][P,2]NG |
| 12_CDS32  cell surface glycoprotein precursor |  | 76-104 | 14 | 2.07 |  |
| 4_CDS35  cell surface  glycoprotein precursor | 11 | 70-94 | 12 | 2.08 | [TE,2][TP,2][P,2]NG |
| 4_CDS1 cell surface  glycoprotein | 12 | 41-71 | 10 | 3.10 | TS[TP,4] |
| eHwalsbyi599  probable cell surface  glycoprotein |  | 2795-2838 | 10 | 4.30 |  |
| 4_CDS13  halomucin2 | 13 | 2128-2170 | 10 | 4.30 | T[G,2]VD[DG,2]D |
| 7_CDS9  halomucin2 |  | 1565-1625 | 10 | 6.10 |  |
| 1_CDS31  S-layer protein | 14 | 752-788 | 8 | 4.38 | E[PT,2]ATE |
| 4_CDS8  probable cell  surface glycoprotein | 15 | 4-28 | 8 | 3.00 | [D,2][G,6] |
| 1_CDS12  sugar epimerase/  dehydratase-like protein | 16 | 232-247 | 8 | 2.00 | T[ES,3]E |
| 1_CDS30  cell surface  glycoprotein | 17 | 1191-1205 | 7 | 2.00 | IAVA[L,2]T |
| 6_CDS42  hypothetical  protein | 18 | 36-49 | 7 | 2.00 | VGVFAIL |
| 6_CDS2  cell surface glycoprotein | 19 | 39-55 | 6 | 2.83 | TPTSTP |
| 7_CDS5  cell surface glycoprotein | 20 | 218-250 | 6 | 5.17 | P[TA,2]T |
| eHwalsbyi559 CDS  probable cell surface  glycoprotein |  | 218-250 | 6 | 5.17 |  |
| 9_CDS04  hypothetical protein | 21 | 7-19 | 6 | 2.17 | LTVIVT |
| 9_CDS10  GLUG  domain protein | 22 | 501-512 | 6 | 2.00 | VGLFEA |
| eHwalsbyi559  probable cell  surface glycoprotein | 23 | 2844-2855 | 6 | 2.00 | S[G,5] |
| 1_CDS35  S-layer protein | 24 | 344-354 | 5 | 2.20 | AVGDL |
| 4 _CDS43  S-layer protein |  | 344-354 | 5 | 2.20 |  |
| 5_CDS17  hypothetical  protein |  | 344-354 | 5 | 2.20 |  |
| 6_CDS29  S-layer  protein |  | 344-354 | 5 | 2.20 |  |
| 12_CDS39  S-layer domain  protein |  |  |  |  |  |
| 1_CDS38  RND superfamily  multidrug efflux  system protein | 25 | 405-414 | 5 | 2.00 | IAV[L,2] |
| 5_CDS18  RND superfamily  multidrug efflux  system protein |  | 385-394 | 5 | 2.00 |  |
| 6_CDS32  RND superfamily  multidrug efflux  system protein |  | 407-416 | 5 | 2.00 |  |
| 12_CDS40  RND superfamily  multidrug efflux  system protein |  | 387-396 | 5 | 2.00 |  |
| 4_CDS19  subtilisin like serine protease | 26 | 623-633 | 5 | 2.20 | ITVNT |
| eHwalsbyi559 CDS  subtilisin like serine protease |  | 633-643 | 5 | 2.20 |  |
| 4_CDS35  cell surface  glycoprotein precursor | 27 | 842-851 | 5 | 2.00 | VSGES |
| 5_CDS12  cell surface  glycoprotein precursor |  | 865-874 | 5 | 2.00 |  |
| 12_CDS32  cell surface  glycoprotein precursor |  | 874-883 | 5 | 2.00 |  |
| 5_CDS11  5' nucleotidase | 28 | 18-27 | 5 | 2.00 | LG[A,2]G |
| 12_CDS31  5' nucleotidase |  | 18-27 | 5 | 2.00 |  |
| eHwalsbyi559_CDS  '3'-cyclic-nucleotide  2'-phosphodiesterase |  | 19-28 | 5 | 2.00 |  |
| 5_CDS14  S-layer protein | 29 | 710-719 | 5 | 2.00 | EGTLS |
| 12_CDS34  S-layer_protein |  | 594-603 | 5 | 2.00 |  |
| 6_CDS17  major variable  cell surface protein | 30 | 313-322 | 5 | 2.00 | ITDFT |
| 7_CDS8  Hypothetical protein | 31 | 133-142 | 5 | 2.00 | [R,2]QLP |
| 7_CDS16  ABC type cobalamin/  Iron III siderophore  transport systems  substrate binding protein | 32 | 431-440 | 5 | 2.00 | GEISI |
| 7_CDS18  hypothetical protein |  | 289-298 | 5 | 2.00 |  |
| 9_CDS10  GLUG domain protein | 33 | 517-527 | 5 | 2.20 | TLADV |
| eHwalsbyi559 CDS  probable cell surface  glycoprotein | 34 | 2624-2644 | 5 | 3.80 | E[TP,2] |
| 1_CDS31  S-layer protein | 35 | 788-797 | 4 | 2.50 | S[T,2]G |
| 9_CDS10  GLUG domain protein |  | 195-204 | 4 | 2.50 |  |
| 4_CDS4  Cell surface glycoprotein | 36 | 152-163 | 4 | 3.00 | TATS |
| 7_CDS7  hypothetical protein |  | 165-176 | 4 | 3.00 |  |
| 4_CDS4  Cell surface glycoprotein | 37 | 574-585 | 4 | 3.00 | P[S,3] |
| 5_CDS12  cell surface glycoprotein  precursor | 38 | 223-237 | 4 | 3.75 | TESE |
| 7_CDS15  putative long tail fiber  proximal subunit | 39 | 257-267 | 4 | 2.75 | V[G,2]L |
| eHwalsbyi559_CDS probable cell surface glycoprotein | 40 | 682-691 | 4 | 2.50 | GDQP |
| 1_CDS43  hypothetical protein | 41 | 135-145 | 2 | 5.50 | GR |
| 4_CDS4  cell surface protein | 42 | 478-487 | 2 | 5.00 | NS |
| 4_CDS6  cell surface  glycoprotein | 43 | 39-55 | 2 | 8.50 | TP |
| 4_CDS8  probable cell  surface glycoprotein |  | 209-219 | 2 | 5.50 |  |
| 4_CDS19  subtilisin-like serine protease |  | 526-571 | 2 | 23.00 |  |
| 1 5_CDS1  subtilisin like serine protease |  | 526-583 | 2 | 29.00 |  |
| 6_CDS1  cell surface glycoprotein |  | 35-49 | 2 | 7.50 |  |
| 7_CDS9_  halomucin2 |  | 1810-1820 | 2 | 5.50 |  |
| 7_CDS12  cell surface  glycoprotein |  | 41-61 | 2 | 10.50 |  |
| 9_CDS3  cell surface  glycoprotein |  | 39-55 | 2 | 8.50 |  |
| 9_CDS26  subtilisin-like  serine protease |  | 526-557 | 2 | 16.00 |  |
| eHwalsbyi559_CDS subtilisin-like serine protease |  | 526-581 | 2 | 28.00 |  |
| eHwalsbyi559_CDS probable cell surface glycoprotein |  | 3032-3042 | 2 | 5.50 |  |
| 7_CDS8  hypothetical_protein | 44 | 23-33 | 2 | 5.50 | RV |
| 7_CDS19  PKD domain protein | 45 | 96-105 | 2 | 5.00 | DT |
| 12_CDS32  cell surface glycoprotein precursor | 46 | 41-52 | 2 | 6.00 | SG |
