# Supplementary material for: Diversity of the cell-wall associated genomic island of the archaeon Haloquadratum walsbyi
Source: BMC Genomics. 2015 Aug 13;16(1):603. doi: 10.1186/s12864-015-1794-8 (PMC4535781; doi:10.1186/s12864-015-1794-8)
Supplement: Additional file 7: — List of putative recombination events as detected by RDP software. (DOCX 18 kb) [file 12864_2015_1794_MOESM7_ESM.docx]

# Additional file 7. List of putative recombination events as detected by RDP software

|  |  | Breakpoint positions | | | |  |  |  |  |  |  |  |  |  |  |  |
| --- | --- | --- | --- | --- | --- | --- | --- | --- | --- | --- | --- | --- | --- | --- | --- | --- |
|  |  | in recombinant sequence | | in alignment | | parental sequences | | Detection methods | | | | | | | | |
| GI1 name  /number | No. of event  in group | begin | end | begin | end | minor | major | RDP | AD1000- GENECON | Bootscan | Maxchi | Chimaera | SiSscan | PhylPro | LARD | 3Seq |
| 9 | 4 | 5655 | 13240 | 1192682 | 1206553 | HBSQ001 | 1 | 4.31E-72 | 1.71E-70 | 4.55E-33 | 7.66E-34 | 7.66E-33 | 4.03E-48 | NA | NA | 9.70E-74 |
| 9 | 18 | 5905 | 6932 | 1192968 | 1193995 | 5 | HBSQ001 | 4.82E-7 | 1.36E-06 | NA | 5.55E-10 | 2.26E-08 | NA | NA | NA | 6.41E-05 |
| 9 | 1 | 7948 | 24386 | 1195084 | 1229780 | 1 | 5 | 3.25E-99 | 6.57E-96 | NA | 3.20E-29 | 1.49E-20 | 4.35E-73 | NA | NA | 2.96E-116 |
| 6 | 14 | 28357 | 6511 | 1229195 | 1176930 | C23 | HBSQ001 | 3.43E-22 | NA | NA | 2.74E-11 | 2.06E-11 | 3.59E-16 | NA | NA | 1.26E-20 |
| 6 | 15 | 16103 | 16818 | 1193669 | 1194457 | HBSQ001 | 5 | NA | 9.56E-17 | 5.50E-18 | 2.54E-09 | 9.81E-05 | 7.85E-13 | NA | NA | 2.66E-14 |
| 6 | 1 | 21558 | 23729 | 1208498 | 1223900 | 1 | 5 | 3.25E-99 | 6.57E-93 | NA | 3.20E-29 | 1.49E-20 | 4.35E-73 | NA | NA | 2.96E-116 |
| 4 | 12 | 27515 | 27704 | 1235455 | 1235644 | 6 | 559 | 7.95E-19 | 7.45E-13 | NA | 2.78E-04 | 4.19E-04 | NA | NA | NA | NA |
| 4 | 10 | 28049 | 29379 | 1235989 | 1237319 | 6 | 559 | 4.47E-22 | 1.36E-08 | NA | 8.69E-04 | 1.80E-04 | NA | NA | NA | NA |
| 1 | 26 | 22226 | 22812 | 1222860 | 1223483 | 9 | 7 | 6.31E-18 | NA | NA | 1.04E-09 | 8.97E-11 | NA | NA | NA | 5.25E-03 |
| 1 | 3 | 22981 | 25856 | 1223893 | 1227050 | 5 | 7 | 1.26E-97 | 5.75E-93 | NA | 8.24E-30 | 1.27E-30 | 1.15E-50 | NA | NA | 7.21E-99 |
| 1 | 9 | 26474 | 26729 | 1231207 | 1231468 | 5 | 9 | NA | 1.76E-14 | NA | 2.80E-08 | 6.95E-09 | 2.77E-16 | NA | NA | 1.86E-04 |
| 1 | 7 | 26766 | 29133 | 1231801 | 1236808 | 5 | 559 | 1.10E-30 | 1.46E-38 | 8.83E-04 | 5.99E-25 | 1.20E-18 | 1.81E-48 | NA | NA | 4.71E-10 |
| C23 | 13 | 6867 | 6930 | 1231771 | 1231834 | 7 | 5 | 5.83E-18 | 3.49E-17 | NA | 2.49E-06 | NA | NA | NA | NA | NA |
| HBSQ001 | 1 | 11934 | 17022 | 1194963 | 1223214 | 1 | 5 | 3.24E-99 | 6.57E-93 | NA | 3.20E-29 | 1.49E-20 | 4.35E-73 | NA | NA | 2.96E-116 |
| HBSQ001 | 19 | 17117 | 17475 | 1223346 | 1223900 |  | 7 | 4.68E-10 | NA | NA | 5.91E-09 | 2.96E-06 | NA | NA | NA | 1.39E-03 |
| 5 | 16 | 11035 | 12106 | 1191254 | 1192654 | 9 | C23 | 1.66E-09 | 3.33E-17 | NA | 8.34E-10 | 2.59E-13 | 1.80E-11 | NA | NA | 4.61E-08 |
| 5 | 6 | 18560 | 18705 | 1201171 | 1206607 | HBSQ001 | HBSQ001 | NA | 8.59E-41 | NA | 4.81E-20 | 2.14E-20 | NA | NA | NA | NA |
| 7 | 9 | 1131806 | 1132261 | 1231010 | 1231468 | 5 | 9 | NA | 1.76E-14 | NA | 2.80E-08 | 6.95E-09 | 2.77E-16 | NA | NA | 1.86E-04 |
| 7 | 2 | 1132431 | 1138084 | 1232090 | 1239979 | C23 | 559 | 1.83E-101 | 1.08E-114 | 1.07E-17 | 8.53E-19 | 2.87E-26 | 2.05E-90 | NA | NA | 1.70E-102 |
