# Supplementary material for: Diversity of the cell-wall associated genomic island of the archaeon Haloquadratum walsbyi
Source: BMC Genomics. 2015 Aug 13;16(1):603. doi: 10.1186/s12864-015-1794-8 (PMC4535781; doi:10.1186/s12864-015-1794-8)
Supplement: Additional file 8: — Ka/Ks values for most frequent groups of gene homologs detected in this study. (DOCX 25 kb) [file 12864_2015_1794_MOESM8_ESM.docx]

# Additional file 8. Table S8. Ka/Ks values for most frequent groups of gene homologs detected in this study.

| **Ka/Ks values for each node in the tree on Figure 4 for homologs of**: | | | | | | |
| --- | --- | --- | --- | --- | --- | --- |
| **HQ1193A** | Ka/Ks Branch1 | Ka Branch1 | Ks Branch1 | Ka/Ks Branch2 | Ka Branch2 | Ks Branch2 |
| 1 | 0.2878 | 0.01639289 | 0.05696654 | 0.2991 | 0.01223611 | 0.04091151 |
| 2 | 0.5123 | 0.5574 | 10.880 | 0.4657 | 0.00851196 | 0.01827615 |
| 3 | 0.6209 | 0.03643803 | 0.05869019 | 0.5559 | 0.00899935 | 0.01618848 |
| 4 | 0.4685 | 0.00743133 | 0.01586166 | 0.3673 | 0.00739859 | 0.02014089 |
| 5 | 0.4062 | 0.00748143 | 0.01841824 | 0.3671 | 0.00680463 | 0.01853793 |
| **HQ1194A** |  |  |  |  |  |  |
| 1 | 0.3304 | 0.08787267 | 0.2660 | 11.477 | 0.00935455 | 0.00815046 |
| 2 | 19.586 | 0.01648016 | 0.00841446 | 0.5117 | 0.00336608 | 0.00657832 |
| 3 | 0.6173 | 0.00112513 | 0.00182268 | 0.02374224 | 0.00036482 | 0.01536594 |
| 4 | 0.4854 | 0.00204354 | 0.00420958 | 0.4258 | 0.00207702 | 0.00487793 |
| **HQ1195A** |  |  |  |  |  |  |
| 1 | 0.2648 | 0.02923719 | 0.1104 | 0.3131 | 0.03664720 | 0.1170 |
| 2 | 12.575 | 0.5081 | 0.4041 | 0.6447 | 0.3482 | 0.5401 |
| 3 | 94.885 | 0.00948849 | 1,00E-10 | 0.2562 | 0.00589405 | 0.02300970 |
| 4 | 0.6070 | 0.3634 | 0.5986 | 0.6016 | 0.2769 | 0.4603 |
| **HQ1197A (hmu2)** | |  |  |  |  |  |
| 1 | 0.7106 | 0.4981 | 0.7010 | 0.7127 | 0.01181624 | 0.01657932 |
| 2 | 0.3778 | 0.00523816 | 0.01386512 | 0.3262 | 0.00399436 | 0.01224496 |
| 3 | 0.7839 | 0.1459 | 0.1861 | 0.7782 | 0.1181 | 0.1517 |
| **HQ1200A (subtilisin-like serine protease)** | |  |  |  |  |  |
| 1 | 0.2282 | 0.01639786 | 0.07186229 | 0.1663 | 0.00799821 | 0.04810685 |
| 2 | 0.4844 | 0.01927188 | 0.03978208 | 0.1776 | 0.00919185 | 0.05175136 |
| 3 | 0.2340 | 0.01131735 | 0.04835943 | 0.2351 | 0.01303995 | 0.05545504 |
| **HQ1205A (major variable surface protein)** | |  |  |  |  |  |
| 1 | 0.8842 | 0.4561 | 0.5158 | 0.6912 | 0.3349 | 0.4845 |
| 2 | 0.5708 | 0.2208 | 0.3868 | 0.8886 | 0.3633 | 0.4089 |
| 3 | 0.7100 | 0.2154 | 0.3033 | 0.6340 | 0.1796 | 0.2832 |
| 4 | 0.5344 | 0.2070 | 0.3874 | 0.5015 | 0.1898 | 0.3785 |
| **HQ1206A (cell surface glycoprotein)** | |  |  |  |  |  |
| 1 | 0.5562 | 0.3109 | 0.5590 | 0.5357 | 0.2982 | 0.5567 |
| 2 | 0.5007 | 0.3712 | 0.7414 | 0.3367 | 0.02993523 | 0.08891534 |
| 3 | 21.009 | 0.00210085 | 0.00089493 | 0.4606 | 0.00065167 | 0.00141474 |
| 4 | 0.2794 | 0.03421774 | 0.1225 | 0.2738 | 0.02996365 | 0.1095 |
| **HQ1207A (S-layer protein)** | |  |  |  |  |  |
| 1 | 0.6764 | 0.08225077 | 0.1216 | 0.4731 | 0.03895846 | 0.08234170 |
| 2 | 0.4613 | 0.07068190 | 0.1532 | 0.4170 | 0.05172039 | 0.1240 |
| 3 | 0.4010 | 0.1465 | 0.3655 | 0.4183 | 0.1577 | 0.3769 |
| 4 | 0.4153 | 0.00647546 | 0.01559346 | 0.5338 | 0.00802462 | 0.01503301 |
| 5 | 0.4015 | 0.1305 | 0.3251 | 0.3567 | 0.1163 | 0.3261 |
